# Cutting Force—Vibration Interactions in Precise—and Micromilling Processes: A Critical Review on Prediction Methods

**DOI:** 10.3390/ma18153539

**Published:** 2025-07-28

**Authors:** Szymon Wojciechowski, Marcin Suszyński, Rafał Talar, Vit Černohlávek, Jan Štěrba

**Affiliations:** 1 Faculty of Mechanical Engineering, Poznan University of Technology, Piotrowo 3, 60-965 Poznan, Poland; marcin.suszynski@put.poznan.pl (M.S.); rafal.talar@put.poznan.pl (R.T.); 2 Department of Automobile and Manufacturing Technologies, Faculty of Manufacturing Technologies with a Seat in Presov, Technical University of Kosice, 31 Sturova St., 080 01 Presov, Slovakia; 3 Faculty of Mechanical Engineering, Jan Evangelista Purkyně University in Ústí nad Labem, Pasteurova 1, 400 96 Ústí nad Labem, Czech Republic; vit.cernohlavek@ujep.cz (V.Č.); jan.sterba@ujep.cz (J.Š.)

**Keywords:** precise machining, micromilling, cutting forces, vibrations

## Abstract

In recent years, much research has been devoted to the evaluation of physical phenomena and the technological effects of precise and micromilling processes. However, the available current literature lacks synthetic work covering the current state of the art regarding cutting force–tool displacement interactions in precise and micromilling manufacturing systems. Therefore, this literature review aims to fill this research gap and focuses on the critical literature review regarding the current state of the art within the prediction methods of cutting forces and machining system’s displacements/vibrations during precise and micromilling techniques. In the first part, a currently available cutting force, as well as the static and dynamic machining system displacement models applied in precise and micromilling conditions are presented. In the next stage, a relationship between the geometrical elements of cut and generated cutting forces and tool displacements are discussed, based on the recent literature. A subsequent part concerns the formulation of the generalized analytical models for a prediction of cutting forces and vibrations during precise and micromilling conditions. In the last stage, the conclusions and outlook are formulated based on the conducted analysis of the literature. In this context, this paper constitutes a synthetic work presenting current trends in the prediction of precise milling and micromilling mechanics.

## 1. Introduction

Precise milling and micromilling are currently leading techniques for the accurate manufacturing of precise parts made of metals and hard-to-cut materials. The fundamental goal of these techniques is to obtain manufactured parts with strict requirements towards the machined surface finish (mainly the dimensional accuracy, as well as the surface roughness and surface waviness) [1,2,3,4]. However, the machined surface quality is substantially affected by machining loads (i.e., cutting forces) [5,6,7] and the resulting vibrations in the machining system [8,9,10,11,12,13]. 

The total cutting force components generated during machining are the effect of the interaction between the workpiece material and the cutting edge, as well as the kinematics of the process. These values have a complex effect on all physical phenomena and technological effects accompanying cutting. Therefore, cutting forces are treated as a physical machinability indicator, providing important information about the course of the machining process. An increase in the force value usually leads to accelerated wear of the cutting edge, an increase in the tool deflection value, and the formation of a built-up edge on the cutting edge [14,15,16,17].

Becze et al. [18] and Twardowski et al. [19] observed that a significant wear mechanism of solid carbide milling cutters during high-speed machining of hardened steel (HSM) is the chipping of the cutting edges caused by excessive force values. Lopez de Lacalle et al. [20] prove that the force values have a significant effect on the dimensional deviation of the curvilinear surface machined with a spherical cutter. The highest values of the normal force generated during machining are correlated with the areas of the highest dimensional deviation values. This is directly related to the proportionality of the force to the tool deflection. In addition to the dimensional deviation, the forces affect the machined surface roughness. According to Wojciechowski et al. [21], the use of optimal milling parameters, minimizing the values of the total force components during precise milling of hardened steel, allows for the reduction in the values of the surface roughness parameters *Sa* and *Sz*.

Due to the fundamental importance of forces in the machining process, intensive research has been conducted for many years to develop reliable models of the total force components for various cutting methods and diverse input parameters. Conventionally, these models can be divided into analytical [22,23,24,25,26] and experimental [27,28,29,30,31,32] models. These models are used to predict forces for various cutting methods, workpiece and tool materials, and machining parameters. However, they mainly concern conventional machining processes, carried out in the range of uncut chip thickness significantly higher than a cutting edge radius *h* >> *r_n_*. In precision cutting conditions, in which the uncut chip thickness *h* corresponds to the order of magnitude of the cutting edge radius *r_n_*, the conventional cutting theory, based on a sharp cutting edge, is not applicable. This is caused by the intensive effect of the negative rake angle on the chip formation phenomenon. As a result, the material shearing may not occur along a single shear plane (as in conventional cutting) but may be distributed continuously along the rounded cutting edge [33]. In addition, the elastic and elastic–plastic deformation phenomena occurring for cutting with uncut chip thicknesses lower than a minimum uncut chip thickness value *h* < *h_min_* contribute to the generation of ploughing forces, which have a significant share in generating the resultant force value. Therefore, according to Kim et al. [34], conventional force models should not be used when *f_z_*/*r_n_* < 0.1. An alternative to conventional models may be models, which take into account the minimum uncut chip thickness, geometrical errors of machining system, as well as complex thermomechanical phenomena occurring between the workpiece and the cutting edge [35,36,37,38,39,40,41].

The subsequent phenomena occurring during precise and micromilling processes, which can be directly correlated with cutting forces are displacements and vibrations located in the machine–toolholder–tool–workpiece system. These displacements/vibrations are an indispensable phenomenon accompanying the cutting process, but they are highly undesirable. They fundamentally affect the geometric indicators of a machined surface integrity (e.g., surface roughness, surface waviness, shape errors) [42,43,44,45,46,47,48,49,50,51], the tool wear intensity [52], and also, under certain conditions, the loss of dynamic stability of the process due to the occurrence of self-excited vibrations [53,54,55,56,57,58]. Therefore, the issue of research and modeling the relationship between displacements in the machining system and the input parameters of the process is currently a very important research problem, mainly in relation to conventional machining. In the case of precision cutting, this subject becomes even more important. This is mainly due to the specificity of precision and micromachining technologies, which are based on the micrometric selection of values of the geometric elements of cut (i.e., uncut chip thickness and width of cut). As a result, displacements of the machining system elements of the order of several micrometers significantly affect the physical phenomena and technological effects of the process. Therefore, identifying the sources of displacements is of key importance in terms of recognizing and understanding the phenomena related to their occurrence. It should be emphasized that a more complete understanding of these issues may enable the selection of input parameters that affect the reduction in the displacement values in the machining system. 

**Figure 1:**
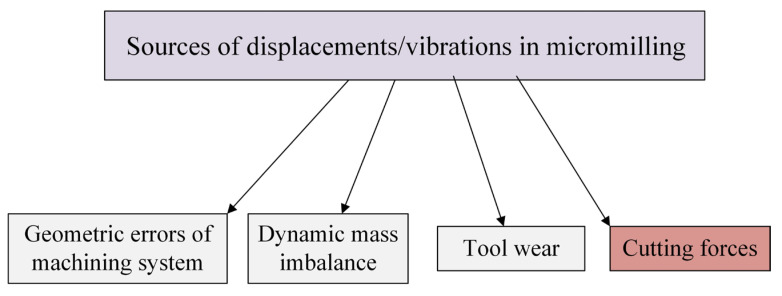
Sources of displacements/vibrations in machining.

Figure 1 shows the main sources of displacements of the machining system elements during machining. One of the main sources are geometric errors of the machining system. They include errors in the manufacture of the tool, holder and/or machine (e.g., tilt or eccentricity of the geometric tool axis in relation to the spindle rotation axis, errors in the manufacture and setting of cutting inserts, as well as errors in tool sockets). Displacements in the machining system can also be caused by errors in the machine axis [59]. Another source of displacements is the dynamic imbalance of the machining system elements. This problem becomes particularly important in processes carried out in the range of high spindle rotational speeds (e.g., high speed machining—HSM technique or micromilling). This results from the fact that dynamic imbalance leads to the generation of a centrifugal force proportional to the square of the rotational speed, which in turn leads to the generation of tool deflection. According to the works [60,61], the main causes of unbalance of the machining system elements include inaccuracy of shape and size, material inhomogeneity, asymmetric structure (e.g., radial mounting screws in some holders), as well as the phenomenon of radial run-out.

The tool wear that progresses during the machining process can be a source of displacements in the machining system. This phenomenon manifests itself in a change in the shape and/or loss of the tool working part mass, which consequently affects the retraction of the cutting edge and changes in the actual values of the geometric parameters of cut. It should be emphasized that in the case of multi-teethed tools (e.g., milling cutters), wear usually does not progress evenly for all cutting edges. This leads to the generation of force oscillations during machining, contributing to the variability of the tool deflection. Consequently, it can induce the deterioration of a machined surface finish [62,63,64,65,66,67,68,69]. 

Another source of displacements during machining is the heat resulting from mechanical work in the cutting zone. This phenomenon significantly affects the thermal expansion of the tool or workpiece. Klocke et al. [70] found that during the turning of 1045 steel, in the range of feed *f* = 0.1 mm/rev and cutting speed *v_c_* = 100 m/min, the radial expansion of the workpiece reaches about 20 µm. According to Creighton et al. [71], in case of high-speed micro-milling spindles, thermally induced displacement in the tool–workpiece interface can be reduced from 6 microns to less than 1 micron with appropriate compensation models.

Despite the significant role of the above-mentioned sources, the forces occurring during cutting are one of the most important factors determining the deflections of the machining system elements. The deflection values and the nature of their variability depend on the values and frequencies of the total force components, as well as on the mechanical properties of the machining system elements. Therefore, the selection of optimal machining parameters, as well as the appropriate design of the machining system elements, can contribute to minimizing the values of the forces generated during cutting, and thus also the deflection. Therefore, a lot of research has been devoted to modeling the tool deflection during cutting. In the most general sense, these models can be divided into static and dynamic.

In the proposed work, the authors investigate the state-of-the-art regarding a cutting force and displacement/vibration prediction methods, as well as their interactions in the precise and micromilling processes. The key objectives of this work are as follows:

- Presentation of the recent analytical cutting force and displacement prediction methods in precise and micromilling processes;
- Indication of a key factors, characteristic for a precise and micromilling processes, which affect the geometric parameters of cut and thus the cutting forces and displacements in the machining system;
- Formulation of the generalized cutting forces, displacements/vibrations models, based on the analysis of the recent and critical literature;
- Identification of challenges and future directions in cutting force and displacement prediction methods dedicated to precise and micromilling processes.

## 2. Cutting Force Models Applied in Precise Milling and Micromilling

In order to estimate the forces in a precise and micromilling, approaches based on the modification of mechanistic models are used, due to the range *h* ≤ *h_min_*, as well as analytical models (based on the so-called slip zone theory) and hybrid models (concerning the use of FEM methods and mechanistic/analytical models). 

**Figure 2:**
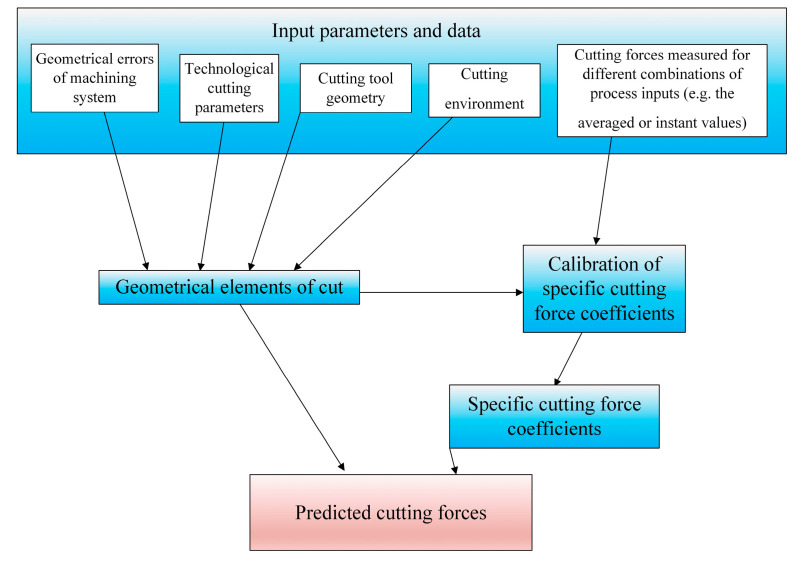
The schematic diagram of cutting force prediction based on the mechanistic model.

In the mechanistic approach for precision milling, the modified Lee–Altintas model is used [31], which assumes the proportionality of the instant cutting forces to the geometrical elements of cut and specific force coefficients determined in a series of the calibration tests (Figure 2). 

In its basic form, this model takes into account the influence of shearing and chip formation, as well as phenomena occurring along the active cutting edge, related to frictional contact phenomena between the unrounded cutting edge (*r_n_* = 0) and the workpiece (so-called edge forces). However, in the form intended for precision cutting, the model assumes that *r_n_* > 0 and takes into account the presence of ploughing forces when *h* < *h_min_*. According to the references [72,73,74,75], the total force generated during precision milling can be decomposed into components in the tool system acting on the elementary segment of the cutting edge. This relationship can be described by the equations:

(1) $$\left\{\begin{array}{l} dF_{t}=\left[K_{tc}h_{z}+K_{te}\right]dz\\ dF_{r}=\left[K_{rc}h_{z}+K_{re}\right]dz,\;\;h>h_{\min}\\ dF_{a}=\left[K_{ac}h_{z}+K_{ae}\right]dz \end{array}\right.$$

(2) $$\left\{\begin{array}{l} dF_{t}=\left[K_{tp}A_{pl}+K_{te}\right]dz\\ dF_{r}=\left[K_{rp}A_{pl}+K_{re}\right]dz,\;\;h\leq h_{\min}\\ dF_{a}=\left[K_{ap}A_{pl}+K_{ae}\right]dz \end{array}\right.$$

where

*F_t_*, *F_r_*, *F_a_*—cutting force components, tangential, radial and binormal components,
*K_te_*, *K_re_*, *K_ae_*—specific edge cutting force coefficients,
*K_tc_*, *K_rc_*, *K_ac_*—specific shearing cutting force coefficients,
*K_tp_*, *K_rp_*, *K_ap_*—specific ploughing cutting force coefficients,
*h_z_*—uncut chip thickness per 1 tooth,
d*z*—elementary length of cutting edge segment,
*A*_pl_—ploughing area.

Equations (1) and (2) show that in order to estimate the cutting force components, it is necessary to determine the values of the geometric parameters of the cut (*h_z_*, d*z*), determine the value of the ploughing area *A*_pl_, and also the proportionality coefficients (*K_te_*, *K_re_*, *K_ae_*, *K_tc_*, *K_rc_*, *K_ac_*, *K_tp_*, *K_rp_*, *K_ap_*). The ploughing area determines the area that is not cut as a result of the chip shearing phenomenon but is pressed under the flank face of the tool. Therefore, in order to determine the value of *A*_pl_, it is necessary to know the values of the cutting edge radius *r_n_*, the flank angle *α_n_* and the elastic recovery *Δs* of the workpiece. Experimental or analytical methods can be used to determine the values of the proportionality coefficients. Experimental methods are based on measuring the forces during cutting, then relating the measured values to the equations of the formulated force model and finally transforming these equations with respect to the proportionality coefficients. To that end, one can use approaches that employ orthogonal cutting tests using a transformation for non-orthogonal cutting [76] or calibration of the coefficients in a series of experiments [77,78,79,80,81]. During the calibration of the proportionality coefficients, many cutting trials are carried out with variable cutting parameters for specific combinations of workpiece and cutting tools. As a result, the proportionality coefficients can be identified:

- by fitting an approximating curve to the experimental mean values [82,83,84] or instantaneous values of the force [85,86];
- by formulating an object function expressed as the difference between the measured and calculated force [87];
- by using a method based on Bayesian inference.

The proportionality coefficients can also be determined using analytical methods that do not require time-consuming and costly experimental studies but are based on the knowledge of certain thermomechanical parameters that characterize the cutting process. The authors of [88,89,90] proposed the use of the non-orthogonal model to estimate the proportionality coefficients related to shearing. The mathematical model of the proportionality coefficients, based on this approach, is expressed by the following equations:

(3) $$\begin{array}{l} K_{tc}=\frac{\sigma \cdot \cos(\Theta_{n}-\gamma_{n})+\mathrm{tg}\eta_{c}\cdot \sin\Theta_{n}\cdot \mathrm{tg}\;\lambda_{s}}{\sin\Phi \sqrt{\cos^{2}(\Phi +\Theta_{n}-\gamma_{n})+{\mathrm{tg}}^{2}\;\eta_{c}\cdot \sin^{2}\Theta_{n}}}\\ K_{rc}=\frac{\sigma \cdot \sin(\Theta_{n}-\gamma_{n})}{\sin\Phi \cdot \cos\lambda_{s}\sqrt{\cos^{2}(\Phi +\Theta_{n}-\gamma_{n})+{\mathrm{tg}}^{2}\;\eta_{c}\cdot \sin^{2}\Theta_{n}}}\\ K_{ac}=\frac{\sigma \cdot \cos(\Theta_{n}-\gamma_{n})\cdot \mathrm{tg}\;\lambda_{s}-\mathrm{tg}\;\eta_{c}\cdot \sin\Theta_{n}}{\sin\Phi \sqrt{\cos^{2}(\Phi +\Theta_{n}-\gamma_{n})+{\mathrm{tg}}^{2}\;\eta_{c}\cdot \sin^{2}\Theta_{n}}} \end{array}$$

The calculation of Equation (3) requires knowledge of the slip stress *σ*, the chip friction angle on the rake face *Θ_n_*, the chip flow angle *η_c_* and the shear angle *Φ*. 

The Abdelmoneim–Scrutton model [91] can be used to estimate the specific edge cutting force coefficients, which was developed for cutting with a tool equipped with a large radius of the cutting edge at small depths of cut. This model takes into account the friction forces caused by ploughing, occurring in the range *h* < *h_min_*, and is expressed by the following equations:

(4) $$\begin{array}{l} K_{te}=r_{n}\sigma \left(\frac{2\beta_{\mathrm{kr}}}{\cos\beta_{\mathrm{kr}}}+\pi \sin\beta_{\mathrm{kr}}\;\mathrm{tg}\;\beta_{\mathrm{kr}}\right)\\ K_{re}=r_{n}\sigma \left(2\sqrt{3}\sin\beta_{\mathrm{kr}}\right)\\ K_{ae}=K_{te}\sin\lambda_{s} \end{array}$$

Alternatively, the specific edge coefficients *K_te_*, *K_re_* can be estimated using the analytical slip zone model formulated by Waldorf et al. [92] according to the equations:

(5) $$\begin{array}{l} K_{te}=\left(\begin{array}{l} \cos\;\left(2\Phi_{1}\right)\cos\;\left(\Phi -\Phi_{2}+\Phi_{1}\right)+\\ +\left(1+2\Phi_{3}+2\Phi_{2}+\sin\left(2\Phi_{1}\right)\right)\sin\left(\Phi -\Phi_{2}+\Phi_{1}\right) \end{array}\right)\frac{\sigma \cdot l_{sh}}{\sin\Phi_{1}}\\ K_{re}=\left(\begin{array}{l} \left(1+2\Phi_{3}+2\Phi_{2}+\sin\left(2\Phi_{1}\right)\right)\sin\left(\Phi -\Phi_{2}+\Phi_{1}\right)-\\ -\cos\;\left(2\Phi_{1}\right)\sin\left(\Phi -\Phi_{2}+\Phi_{1}\right) \end{array}\right)\frac{\sigma \cdot l_{sh}}{\sin\Phi_{1}} \end{array}$$

where 

*Φ*_1_, *Φ*_2_, *Φ*_3_—angles characterizing the slip zone.

The method of determining the angles characterizing the slip zone (*Φ*_1_, *Φ*_2_, *Φ*_3_) and the length of the slip zone *l_sh_* is described in detail in work [92]. 

Jun et al. [93] proposed an analytical force model for micro-milling based on the slip zone theory. This model is an extension of the approach developed by Fang [94], which uses the determination of slip lines during orthogonal cutting with a rounded cutting edge radius. The model of Jun et al. [93] takes into account the occurrence of the so-called dead metal zone, located in the area of the rounded cutting edge radius caused by the ploughing phenomenon. In order to model the process of constituting the dead metal zone, the following assumptions were made:

- the workpiece material flowing onto the rounded cutting edge tends to move in the direction determined by the effective rake angle,
- in order to ensure easy material flow, the front section of the dead zone is tangential to the cutting edge.

Taking into account the relationships between the rounded cutting edge, the dead zone geometry and the slip zones, the elementary shear forces can be determined according to the equations:

(6) $$\begin{array}{l} dF_{tc}=\sigma \cdot dz\left[\left(\cos\Phi +a_{1}\sin\Phi \right)l_{ED}+\left(\cos\;\left(2\Phi_{2}\right)\sin\gamma_{ne}+a_{2}\cos\gamma_{ne}\right)l_{AD}\right]\\ dF_{rc}=\sigma \cdot dz\left[\left(a_{1}\cos\Phi -\sin\Phi \right)l_{ED}+\left(\cos\;\left(2\Phi_{2}\right)\cos\gamma_{ne}-a_{2}\sin\gamma_{ne}\right)l_{AD}\right] \end{array}$$

where

*a*_1_, *a*_2_—coefficients taking into account the relationship between the tool and the slip zone,
*l_AD_*—length of the slip zone relative to the frontal section of the dead metal zone.

The elementary ploughing forces are determined based on the relationship:

(7) $$\begin{array}{l} dF_{tp}=\sigma \cdot dz\left[\cos\;\left(2\Phi_{1}\right)\cos\Phi_{3}+a_{1}\sin\Phi_{3}\right]l_{AD}\\ dF_{rp}=\sigma \cdot dz\left[a_{1}\cos\Phi_{3}-\cos\;\left(2\Phi_{1}\right)\sin\Phi_{3}\right]l_{AD} \end{array}$$

The elementary tangential and radial forces, taking into account both the shear and ploughing phenomena, are expressed in the following form:

(8) $$\begin{array}{l} dF_{t}=dF_{tc}+dF_{tp}\\ dF_{r}=dF_{rc}+dF_{rp} \end{array}$$

**Figure 3:**
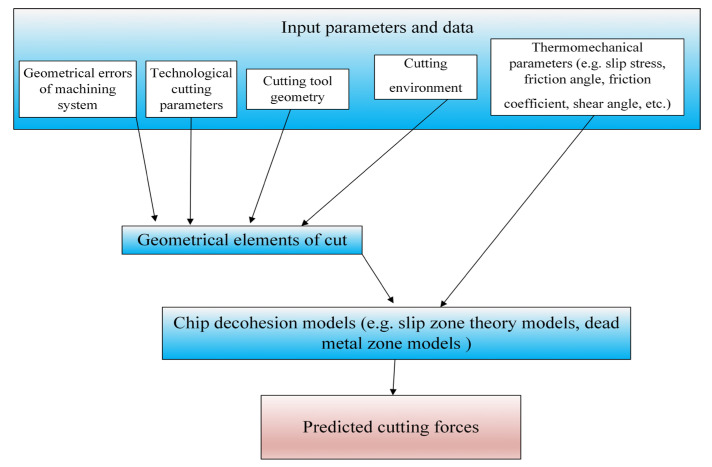
The schematic diagram of cutting force prediction based on analytical model.

A certain limitation of the analytical approaches is the need to determine the values of many thermomechanical coefficients characterizing the model (e.g., shear angles, friction, length of slip zones, etc. See Figure 3). 

**Figure 4:**
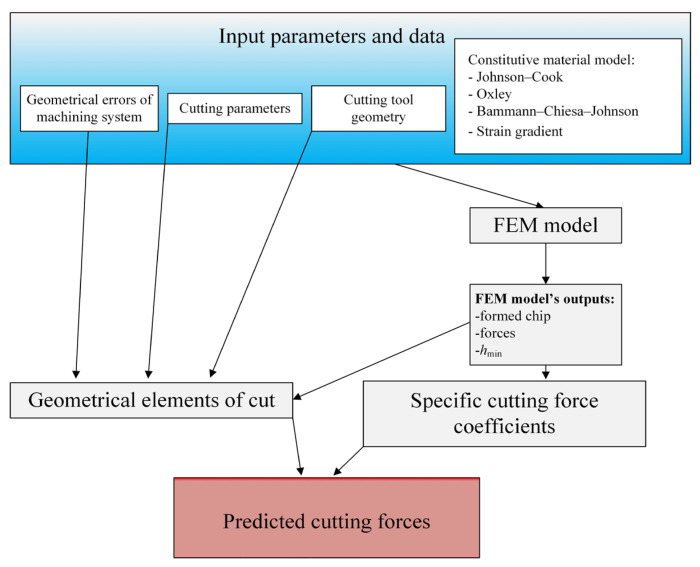
The schematic diagram of cutting force prediction based on a hybrid model.

These values have not been yet identified for all the workpiece materials applied in machining operations. In order to simplify the calculation procedure of forces, hybrid approaches are used, based on a combination of numerical modeling (FEM) with mechanistic or analytical models (Figure 4).

At the first stage of force modeling based on hybrid models, the input parameters of the process are identified (similarly to the mechanistic and analytical approaches), including the selection of cutting parameters and cutting tool geometry. At this stage, an appropriate constitutive model of the workpiece material is also selected, necessary for the development of the FEM model of the cutting process. It should be emphasized that when selecting the constitutive model, both the type of the workpiece material and the complexity of its structure should be taken into account. In the next stage, the FEM model of the process is generated using appropriate software (e.g., ABAQUS). As a result of modeling, the output values are obtained (e.g., kinematic–geometric model of the formed chip, distribution of forces along the cutting edge, minimum uncut chip thickness) that allow for determining the coefficients in the force model. It should be noted that, according to the literature, different forms of force models are used. Afazov et al. [95,96,97] proposed two-component exponential equations that take into account the uncut chip thickness and the cutting speed:

(9) $$\begin{array}{l} F_{t}=\left(a_{t1}\cdot v_{c}^{a_{t2}}\right)\;\left(1-e^{a_{t3}h}\right)\;\;+\left(a_{t4}\cdot v_{c}+a_{t5}\right)\;\left(1-e^{a_{t6}h}\right)\\ F_{r}=\left(a_{r1}\cdot v_{c}^{a_{r2}}\right)\;\left(1-e^{a_{r3}h}\right)\;\;+\left(a_{r4}\cdot v_{c}+a_{r5}\right)\;\left(1-e^{a_{r6}h}\right) \end{array}$$

where

*a_t_*_1_–*a_t_*_6_—proportionality coefficients for the tangential force, determined based on the FEM model,
*a_r_*_1_–*a_r_*_6_—proportionality coefficients for radial force, determined based on the FEM model.

The model of Afazov et al. [95,96,97] was applied to estimate the forces during cylindrical micro-milling of 4340 steel. Li and Wu [2] and Jin and Altintas [98] used a model assuming that the tangential and radial forces are proportional to the cross-sectional area of cut and the specific cutting force coefficients. In the cutting force model applied by authors of the work [2], the specific cutting force coefficients were determined as nonlinear functions of the uncut chip thickness. The authors of the work [98] extended the nonlinear model of specific cutting force coefficients to include the influence of the cutting edge radius. Lai et al. [99] formulated force equations based on the analytical model of the slip line developed by Waldorf [92]. 

**Figure 5:**
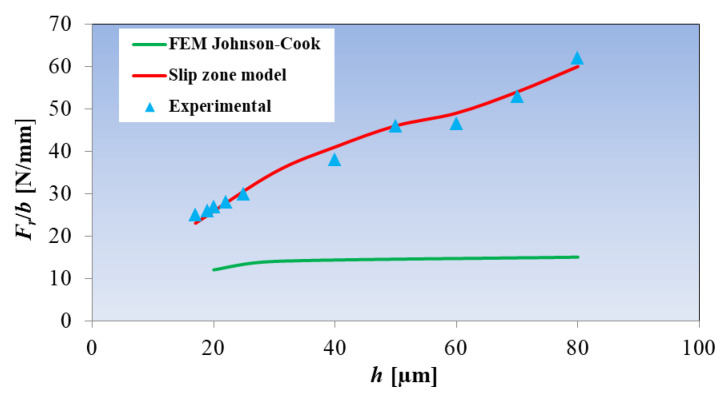
The influence of the uncut chip thickness on radial specific cutting force during micromilling. Developed on the basis of [98,100].

**Figure 6:**
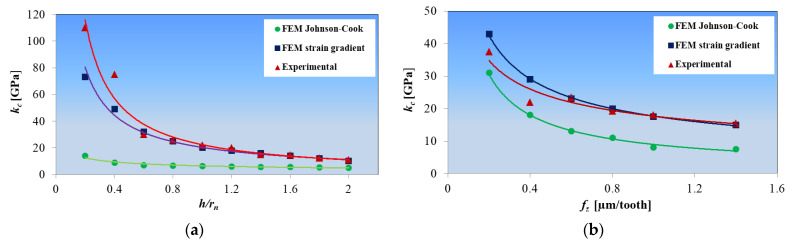
Comparison of the measured and modeled specific cutting force coefficients based on different hybrid force models during micromilling of the following: (**a**) hardened H13 steel, (**b**) copper. Developed on the basis of [99,104].

The last stage of modeling consists of generating instantaneous values of the total force components using the adapted force model, taking into account the input parameters of the machining process and the output values from the FEM model (Figure 4). It should be emphasized that the accuracy of the hybrid model depends to a large extent on the adapted constitutive model of the workpiece material (Figure 5 and Figure 6).

The research conducted by Jin and Altintas [98] shows that the values of radial specific cutting force coefficients estimated using the hybrid model (applying the Johnson–Cook model) are significantly lower (Figure 5) than the values measured and modeled on the basis of the analytical model of the slip zone [100]. According to the authors [98], the reason for the underestimation of the *F_t_*/*b* parameter value is the assumption of a constant value of stresses caused by friction in the FEM model in the contact zone between the tool and the workpiece material. As a consequence, the effects of temperature, size and material deformation rate are not included in the force model. Another reason for the limited accuracy of constitutive models is the omission of the size effect, which affects the intensive hardening of the machined workpiece in the range of small values of the uncut chip thickness *h*. According to Karpat [101], the reasons for this phenomenon are as follows:

- A decrease in the number of microstructure defects with a decrease in *h*;
- An increase in the material deformation rate in the main slip zone;
- The effect of material softening caused by the effect of high temperature;
- The effect of the plastic deformation gradient in the deformation zones, in the range of small uncut chip thicknesses.

Therefore, the authors of [102,103] proposed a constitutive model of the plastic strain gradient, which is an extension of the Johnson–Cook equation. The developed approach takes into account the hardening of the workpiece material in the range of small *h* values. 

Based on the research results of the authors of [99,104] concerning micro-milling of hardened H13 steel and copper, it is confirmed that the use of the constitutive model of the strain gradient in the hybrid force model significantly increases the accuracy of the estimation of specific cutting force coefficients in relation to the results obtained for the approach based on the traditional Johnson–Cook model (Figure 6).

## 3. Characteristics of Relations Between Geometrical Elements of Cut and Cutting Forces

The forces generated during machining depend on the values of the geometric parameters of cut, i.e., uncut chip thickness *h* and uncut chip width *b*. In conventional machining, the selected cutting parameters are significantly greater than the values of the geometric errors of the machining system and the tool deflection caused by the interaction of the total cutting force components. Therefore, these factors are not taken into account when assessing the dynamics of the process. However, the above simplification is not acceptable in the case of precision machining, in which the values of the machining system errors and the tool deflection correspond to the order of magnitude of the geometric parameters of cut [105]. The phenomenon of elastic recovery, determined by the mechanical properties of the workpiece material and the non-zero cutting edge radius, additionally affects the actual values of the volume of the material removed [106]. Therefore, accurate force models developed for precision machining processes should take into account the above-mentioned factors in mathematical expressions describing the geometric parameters of cut. In the conventional milling process, it is assumed that the cutting edge trajectory is circular. In this way, the instantaneous uncut chip thickness per tooth depends on the instantaneous working angle *φ* and the feed per tooth *f_z_* and for peripheral milling is expressed in the form [107]:

(10) $$h_{z}(\varphi )=f_{z}\cdot \sin\varphi$$

In real machining conditions, the trajectory of the tool tip movement is described by a trochoid. The simplification of the tool tip movement model can significantly reduce the accuracy of estimating the geometric parameters of the cut in precision machining. Therefore, the authors of [108] proposed an equation of the instantaneous uncut chip thickness, based on the trochoidal trajectory, according to the equation:

(11) $$h_{z}(\varphi )=R\left[1-\left(\begin{array}{l} 1-\frac{2f_{z}\cdot \sin\varphi }{R+\left(\frac{f_{z}\cdot z}{2\pi }\right)\cos\varphi }-\frac{2f_{z}^{2}\cdot \cos2\varphi }{{\left(R+\left(\frac{f_{z}\cdot z}{2\pi }\right)\cos\varphi \right)}^{2}}+\\ +\sqrt{\frac{f_{z}^{3}\sin\varphi \cdot \cos^{2}\varphi }{{\left(R+\left(\frac{f_{z}\cdot z}{2\pi }\right)\cos\varphi \right)}^{3}}} \end{array}\right)\right]$$

**Figure 7:**
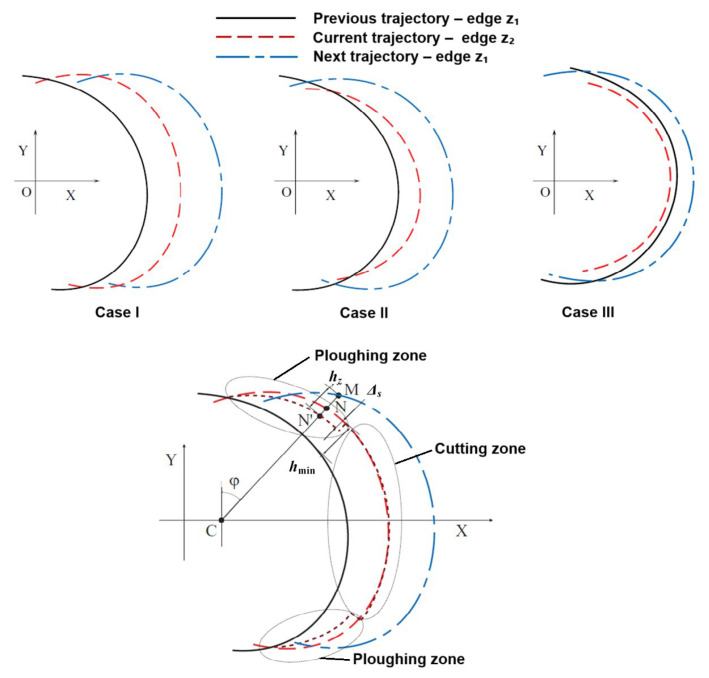
Tool tip trajectory diagram during fully symmetrical micromilling taking into account radial run-out and elastic recovery of the workpiece material (a fine dashed line refers to tool trajectory affected by an elastic recovery of the workpiece). Developed on the basis of Moges et al. [116].

One of the most important factors influencing the variation in the cross-sectional area of cut during precision milling is the phenomenon of the radial run-out *e_r_* of the tool. This factor has been analyzed by many researchers in relation to the estimation of forces in the processes of finishing milling and micro-milling [109,110,111,112]. The radial run-out *e_r_* of the tool is associated with geometric errors of the machining system elements, i.e., errors in the manufacture of the tool, holder, and/or machine tool. According to the works [113,114], the main cause of the radial run-out is the tilt or eccentricity of the geometric axis of the tool relative to the axis of rotation of the spindle. In addition, the radial run-out can be caused by errors in the manufacture and the setting of the cutting inserts, errors in the tool sockets, thermal deformation of the tool and holder, as well as uneven tool wear. Desai et al. [115] showed that under conditions of radial run-out of the tool, the volume of material removed per 1 tooth can be determined not only by the trajectory of the previous cut, but also by the previous trajectories. Consequently, it is necessary to take these trajectories into account when accurately determining the value of the uncut chip thickness *h*. Moges et al. [116] identified three types of interactions between the cutting teeth trajectories in the end milling process using a two-teethed tool (Figure 7).

In case I, the paths of the teeth *z*_1_ and *z*_2_ entering the material interact only with the paths preceding them. This relationship occurs for very small values of the run-out *e_r_* and relatively large feeds *f_z_*. In case II, the next path of the tooth *z*_1_ interacts with both the current path (for the tooth *z*_2_) and the previous path of the tooth *z*_1_. This relationship can be observed in the range of relatively small values of the feeds *f_z_*. Case III is characterized by the interaction of the next path of the tooth *z*_1_ with the previous path for the same tooth *z*_1_, while omitting the current path of the tooth *z*_2_. This case is observed when the run-out value exceeds the feed per tooth (*e_r_* > *f_z_*). This means that one of the teeth does not contact with the workpiece material during a tool pass. 

Another important phenomenon affecting the instantaneous uncut chip thickness and the volume of material removed is the minimum uncut chip thickness. This parameter refers to a specified uncut chip thickness value, at which the initiation of cutting takes place due to the transition from workpiece material’s ploughing to a shearing. In the full symmetric milling process, the tool penetrates the workpiece starting from zeroth uncut chip thickness. Therefore, in the initial phase of tool movement (i.e., in the range, when *h_z_* < *h*_min_) the cutting tool does not cut the workpiece material, but deforms it elastically and elastically–plastically. A certain volume of material is not cut but returns elastically after the tool passes (area marked with dashed lines–Figure 7). This cutter–workpiece–engagement (CWE) section constitutes the so-called ploughing zone. Nevertheless, when the *h_z_* = *h*_min_, the transition from ploughing to shearing takes place, which manifests as initiation of chip formation (see the cutting zone in Figure 7). Subsequently, the chip shearing process continues and, according to full symmetric milling process kinematics, the uncut chip thickness reaches it maximal value at the tool rotation angle *φ* = 90° and then gradually decreases to the *h*_min_ value. This in turn, induces again the transition from chip shearing to a ploughing, manifesting in intense workpiece elastic and plastic deformations (however without chip formation). Finally, the CWE process in a ploughing zone ends during the tool output (i.e., when *h_z_* = 0).

The precise determination of the instantaneous uncut chip thickness should take into account the value of the elastic recovery of the machined material *Δ*_s_. It should be emphasized that the occurrence of the *h*_min_ phenomenon also determines in this way the limits of the tooth mapping in the workpiece material. According to [117], the values of the entry angles *φ_p_* and exit angles *φ_k_* of the tool from the workpiece during full symmetric milling, in the case of the occurrence of the *h*_min_ phenomenon, can be determined from the equations

(12) $$\begin{array}{cc} \varphi_{p}=\arcsin\;\;\left(\frac{h_{\min}}{f_{z}}\right), & \varphi_{k}=\arccos\;\;\left(\frac{h_{\min}}{f_{z}}\right) \end{array}$$

**Figure 8:**
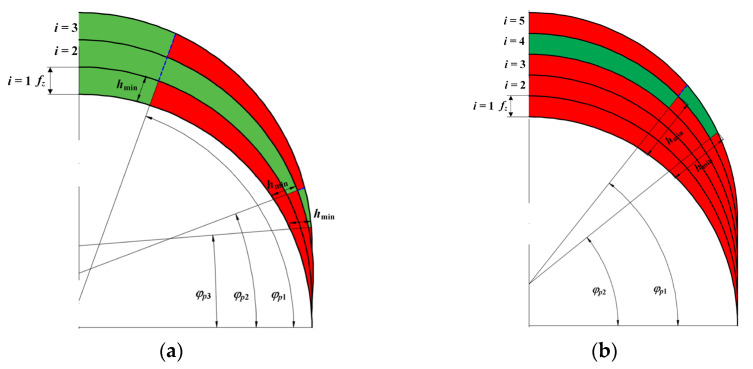
The schematic diagram of the cutting tool tip trajectory during micromilling, concerning the uncut chip thickness accumulation phenomenon (red color refers to a ploughing zone, while a green color to a shearing zone): (**a**) *h*_min_/*f_z_* ≤ 1, (**b**) *h*_min_/*f_z_* > 1. Developed on the basis of [118].

The aforementioned interactions between the minimum uncut chip thickness and the cutter–workpiece–engagement (CWE) conditions have also the influential effect on the so-called uncut chip thickness accumulation mechanism [118]. This phenomenon is usually manifested during machining in a ploughing dominant regime and it concerns the increase in the instantaneous uncut chip thickness in the *i*-th CWE cycle by the value not removed by the cutting tool in the *i* − 1 CWE cycle. Based on the analysis of the geometry of the tool–workpiece mapping, two cases of cutting with the uncut chip thickness accumulation are found, differentiated by the value of the *h*_min_/*f_z_* ratio (Figure 8). 

If *h*_min_/*f_z_* ≤ 1, then the feed per tooth is greater than or equal to the *h*_min_. Therefore, in each CWE cycle there is an area (for *φ* > *φ*_p1_) in which the cutting layer is removed (the area marked in green in Figure 8a). For *φ* < *φ*_p1_, the way the cutting edge interacts with the workpiece depends on the state occurring in the previous CWE cycles. For the case of milling with *h*_min_/*f_z_* > 1 (Figure 8b), the feed per tooth is smaller than the *h*_min_ value. This results in transient occurrence of tooth engagement cycles, in which the workpiece is not transformed into a chip but is ploughed (the area marked in red color in Figure 8b). The appearance of uncut chip thickness accumulation during precision milling can lead to an instant cutting force fluctuations and the nonlinearity of the averaged forces as a function of uncut chip thickness [118].

Significant problems occurring during precision milling with flexible tools (e.g., micro-mills or conventional milling cutters with high slenderness) are also deflections caused by forces generated during machining. According to the authors of [119], these deflections can contribute to the variability of geometric parameters of cut (mainly uncut chip thickness *h_z_*) and to the generation of force fluctuations. In order to determine the tool deflection, it is very often assumed that the milling cutter is an equivalent of an elastic beam fixed in the holder [75]. The components of the total force generated during machining are distributed along the active length of the cutting edge, corresponding to the selected axial depth of cut *a_p_*. In order to simplify the considerations, it can be assumed that the values of forces distributed continuously along the active cutting edge correspond to the value of the concentrated force vector. The vector is applied to the tool in such a way that the obtained bending moment corresponds to the value of the moment obtained in the case of continuous forces. Taking into account the value of the radial run-out and the tool deflection caused by concentrated feed and normal feed forces, the authors of [117] proposed equations of the instantaneous uncut chip thickness for the peripheral micro-milling process:

(13) $$\begin{array}{l} h_{z1}\left(\varphi \right)=f_{z}\cdot \sin\varphi +2\cdot e_{r}\cdot \cos\;\left(\varphi -\lambda_{s}\right)+\frac{1}{k_{s}}\left[\left(1-\frac{\left(1-C_{k}\right)D}{2a_{p}\cdot \mathrm{tg}\;\lambda_{s}}\right)\varphi \right]\\ \;\;\;\;\;\;\;\;\;\;\;\;\;\;\;\;\times \left(\left[-F_{x}\left(z_{1}\right)+F_{x}\left(z_{2}\right)\right]\cdot \sin\varphi +\left[-F_{y}\left(z_{1}\right)+F_{y}\left(z_{2}\right)\right]\cdot \cos\varphi \right)\\ h_{z2}\left(\varphi \right)=f_{z}\cdot \sin\varphi -2\cdot e_{r}\cdot \cos\;\left(\varphi -\lambda_{s}\right)+\frac{1}{k_{s}}\left[\left(1-\frac{\left(1-C_{k}\right)D}{2a_{p}\cdot \mathrm{tg}\;\lambda_{s}}\right)\varphi \right]\\ \;\;\;\;\;\;\;\;\;\;\;\;\;\;\;\;\times \left(\left[F_{x}\left(z_{1}\right)-F_{x}\left(z_{2}\right)\right]\cdot \sin\varphi +\left[F_{y}\left(z_{1}\right)-F_{y}\left(z_{2}\right)\right]\cdot \cos\varphi \right) \end{array}$$

where

*h_z_*_1_(*φ*), *h_z_*_2_(*φ*)—instant uncut chip thickness per first *z*_1_ and second *z*_2_ tooth,
*k_s_*—tool static stiffness coefficient,
*C_k_*—proportionality coefficient of tool deflection,
*F_x_*(*z*_1_), *F_x_*(*z*_2_)—maximal feed normal force values per the first *z*_1_ and second *z*_2_ tooth,
*F_y_*(*z*_1_), *F_y_*(*z*_2_)—maximal feed force values per the first *z*_1_ and second *z*_2_ tooth.

Estimation of the instantaneous values of the uncut chip thickness, in accordance with Equation (13), requires knowledge of the radial run-out value, parameters characterizing the tool stiffness (*k_s_*, *C_k_*) and the maximum values of the forces causing tool deflection.

Research conducted by Jun et al. [120] revealed that the use of a force model that takes into account the geometric errors of the machining system, the elastic recovery of the workpiece material, and the tool deflection (dynamic model) significantly improved the accuracy of force estimation in relation to the results obtained for the so-called static model.

## 4. Evaluation of Displacements and Vibrations in Machining System

### 4.1. Static Models

Static models are most often used to determine the deflection of a cutting tool. They are based on equations describing the static deflection of a single- or double-segment beam mounted in a holder with a static stiffness of the clamping *k_sm_*. In these approaches, the influence of the axial force is ignored due to the relatively high stiffness of the tool in this direction. Considering the milling cutter as a single-segment cylindrical beam, the deflection curve under the influence of the radial force *F_y_*, applied at a distance *L* − *z* from the holder, is described by the equation [76,121]:

(14) $$y_{d}(z)=\frac{F_{y}{\left(L-z\right)}^{2}}{6EI}\left[3L-\left(L-z\right)\right]+\frac{1}{k_{sm}}$$

where

*I*—moment of inertia of the tool cross-section,
*L*—tool overhang.

The model described by Equation (14) can be used to determine the static deflection of cylindrical end mills. In the case of tools with different geometry in the body and shank (e.g., spherical, conical, micro-milling cutters), this approach may be characterized by a significant error resulting from the assumption of only the moment of inertia for the circular cross-section for calculations. Therefore, in order to increase the accuracy of calculations for tools with more complex geometry, Kops and Vo [122] proposed to include in the calculations the so-called equivalent tool radius: *R_e_* = 0.8*R*. However, a more accurate approach is to use a two-segment beam model, taking into account separately the deflection of the tool body *y_nd_* and the shank *y_ud_* [123]. According to the research [42,124], the tool deflection curve for a two-segment beam model, under the influence of the force *F_y_* applied at a distance *L* − *z* from the holder, is described by the equation:

(15) $$\begin{array}{l} y_{d}(z)\;\;\;=y_{ud}+y_{nd}+\varphi_{u}L_{n}=\\ \;\;\;\;\;\;\;\;\;\;\;\;\;\begin{array}{c} =\frac{F_{y}}{6EI_{u}}\left[-{\left(L-L_{n}\right)}^{3}+3{\left(L-L_{n}\right)}^{2}\left(L-z\right)\right]\;\;+ \end{array}\\ \;\;\;\;\;\;\;\;\;\;\;\;\;\begin{array}{c} +\frac{F_{y}}{6EI_{n}}\left[z^{3}-L_{n}^{3}+3L_{n}^{2}\left(L_{n}-z\right)\right]\;\;+ \end{array}\\ \;\;\;\;\;\;\;\;\;\;\;\;\;\begin{array}{c} +\frac{F_{y}}{2EI_{u}}\left[-{\left(L-L_{n}\right)}^{2}+2\left(L-L_{n}\right)\left(L-z\right)\right]L_{n}+\frac{F_{y}}{k_{sm}} \end{array} \end{array}$$

where

*I_u_*—moment of inertia of the cross-section of the tool shank,
*I_n_*—moment of inertia of the cross-section of the tool body,
*L_n_*—length of tool body,
*ϕ_u_*—deflection angle of the tool body.

Static deflection models are usually used to estimate the machined surface topography parameters as surface roughness and surface location errors (SLE). Kim et al. [124] used a two-segment beam model to estimate shape errors during finishing machining of KP4M alloy steel with a ball-nose cutter. A significant effect of the inclination angle on the obtained geometric errors of the machined surface was demonstrated. The same model was also used to predict the deviation of the machined surface profile during end mill machining of Ti6Al4 titanium alloy [76], as well as dimensional errors of a hardened SKD 61 alloy steel after machining with a ball-nose cutter [125]. Hao and Liu [42] used a two-segment beam deflection model to estimate the surface roughness height of 45 steel after machining with a ball-nose cutter. Bo et al. [90] formulated a comprehensive surface topography model that takes into account cutting kinematics, machine errors, and static tool deflection, the estimation of which was based on a two-segment beam model.

The basic advantage of static methods is the relative simplicity of the calculation procedure, based on analytical deflection equations. It should be emphasized that the use of static models in relation to milling is characterized by moderate prediction accuracy. The reason for this is the consideration of tool deflection, which depends on the static mechanical parameters of the tool and holder. In real conditions, the presence of periodically variable or pulsating forces is observed, causing subsequent time-varying deflections, dependent on the dynamic properties of the machining system. Therefore, dynamic deflection models are currently an important alternative to static methods.

### 4.2. Dynamic Models

Dynamic models are used to determine the instantaneous values of cutting tool deflection and instantaneous deformations of the workpiece material (especially thin-walled elements) during machining. The main advantage of these methods is that they take into account the dynamic properties of the machining system, enabling reliable prediction of displacement amplitudes and their frequencies. 

The basis of the vast majority of dynamic models used in precision and micromachining are differential motion equations, formulated in the following form:

(16) $$m\cdot \frac{d^{2}y_{d}}{dt^{2}}+c\cdot \frac{dy_{d}}{dt}+k\cdot y_{d}(t)=F_{y}(t)$$

where

*y_d_*—instant deflection value,
*m*—modal mass,
*c*—damping coefficient,
*k*—dynamic stiffness coefficient,
*F_y_*(*t*)—instant force value in the *Y* direction.

Equation (16) represents a system with one degree of freedom. Models with two degrees of freedom are also very often used to model cutting dynamics. In order to solve the differential equation of motion (16), it is necessary to define the model of the variable force, and also to determine the modal parameters (*m*, *c*, *k*) describing the dynamic properties of the elements of the machining system. From the analysis of the literature, it results that for precision milling, mechanistic [126] and hybrid force models [95] are usually used in differential motion equations. In many studies, the regenerative mechanism is taken into account in order to estimate the dynamic stability of the process. In such cases, the so-called real (dynamic) uncut chip thickness is taken into account in the force expressions, determined by both the kinematic and geometric parameters of the process, as well as the instantaneous values of the tool tip deflection *x_d_*(*t*) and *y_d_*(*t*). According to the work [127], the dynamic uncut chip thickness for end milling can be expressed by the equation:

(17) $$h_{z}(\varphi )=\left[f_{z}+y_{d}(t-T_{z})-y_{d}(t)\right]\sin\varphi +\left[x_{d}(t-T_{z})-x_{d}(t)\right]\cos\varphi$$

where

*y_d_*(*t*), *x_d_*(*t*)—instant tool tip deflections in Y and X directions
*y_d_*(*t* − *T_z_*), *x_d_*(*t* − *T_z_*)—instant tool tip deflections in Y and X directions after time *t* − *T_z_*, where *T_z_* = 60/(*n* ∙ *z*).

The research conducted by the authors of [128,129] shows that the damping forces are a very important factor influencing the dynamic stability of precision milling. Therefore, the expression for the force part in dynamic model should take into account both the shear force and the damping force mentioned above. According to the work of Rahnama et al. [130], the equation of the resultant damping force is formulated in the following form:

(18) $$F_{pd}\left(\varphi \right)=\frac{K_{pd}}{R\cdot n}\cdot \frac{dr_{d}}{dt}$$

where

*K_pd_*—damping coefficient of machining,
*r_d_*(*t*)—instant tool deflection in radial direction.

The next important step in determining the instantaneous deflection values using Equation (17) is to determine the modal parameters, which depend on the mechanical properties and design of the machining system elements. The most popular method for determining the dynamic parameters is the use of the impulse test [131]. This test is based on generating an impulse force excitation using an impulse hammer (equipped with a dynamometer) and measuring the responses of the tool-holder-spindle system in the form of free damped vibrations. A frequency response function (FRF) is obtained, which allows determining the modal parameters. In this method, the vibration sensor is usually mounted on the working part of the tool. In the case of solid carbide micromills, performing an impulse test is significantly inhibited due to the very small size and brittleness of the tool. Therefore, the authors of [132,133] proposed a receptance method which is an integration of the impulse test and FEM simulation. Alternatively, the dynamic parameters of the tool-holder-spindle system can be determined using the inverse dynamics model [134,135]. The main advantage of this method is the elimination of the impulse test. In the approach based on inverse dynamics, the self-excited vibration frequencies and the corresponding critical depth of cut values are determined experimentally. The experiment includes a series of tests in which vibration measurements are made during machining. The obtained results are substituted into the modified equation of the transfer function.

Apart from differential motion equation models, the Euler–Bernoulli and Timoshenko beam equations can be used to predict the dynamic deflection of micromills. Uhlmann and Mahr [136] used the Euler–Bernoulli beam model to evaluate the dynamic stability of the end-milling process of CuZn39Pb1 alloy. The same model was used by Yuan et al. [137] to determine the instantaneous values of the deflection of a ball-nose micromill during machining of 06Cr25Ni20 stainless steel. The Timoshenko beam model was used to estimate the dynamic deflection of a micromill during machining of Al7050 alloy [138] and ferritic–pearlitic steel [93].

The dynamic models presented above are widely used to evaluate physical phenomena and technological effects of micromilling and precision machining. In this context, the precise milling conducted on the industrial robots constitutes the process. The dynamic model of micro-milling cutter deflection proposed by Jun et al. [120] was used to assess the effect of process mechanics on generated vibrations. The developed model concerned micro end milling of ferritic–pearlitic steel and did not take into account geometric errors of the machining system. The authors observed that during machining with feed per tooth values corresponding to the *h_z_* ≤ *h*_min_ range, high vibration amplitude values occur. The conducted analysis of tool displacement power spectral density revealed the presence of a dominant band with a frequency close to the first harmonic of the tool natural frequency. In the case of milling with higher feed per tooth values, related to the *h_z_* > *h*_min_ range, the vibration levels were significantly lower. This phenomenon can be caused by a sudden change in the value of the thrust force as a result of the transition from ploughing to a shearing mode. As a result, this can contribute to the excitation of one or more natural frequencies of the machining system.

The above results have indicated the significance of the problem of dynamic stability loss during precision milling with micro-tools. In conventional machining, loss of dynamic stability is usually correlated with exceeding the critical depth of cut, the value of which ranges from several to several dozen millimeters. In precision machining, very low values of depth of cut and relatively low values of cutting forces occur, which should help maintain process stability. Phenomena characteristic of precision machining, including ploughing and micro-cutting of the workpiece material and inhomogeneity of the structure, can affect the fluctuation of forces [139] leading to the occurrence of self-excited vibrations. This problem becomes of great importance especially in relation to micro-tools (*D* ≤ 1 mm) of very high flexibility. It should be emphasized that due to the very small size of the tools and the associated low values of the acquired force and vibration signals, as well as the relatively high spindle speeds, the identification of self-excited vibrations during micro-milling is a difficult task. In order to recognize the area of self-excited vibrations, the most common spectral analysis is performed of the signals of the total force components or acoustic emission.

**Figure 9:**
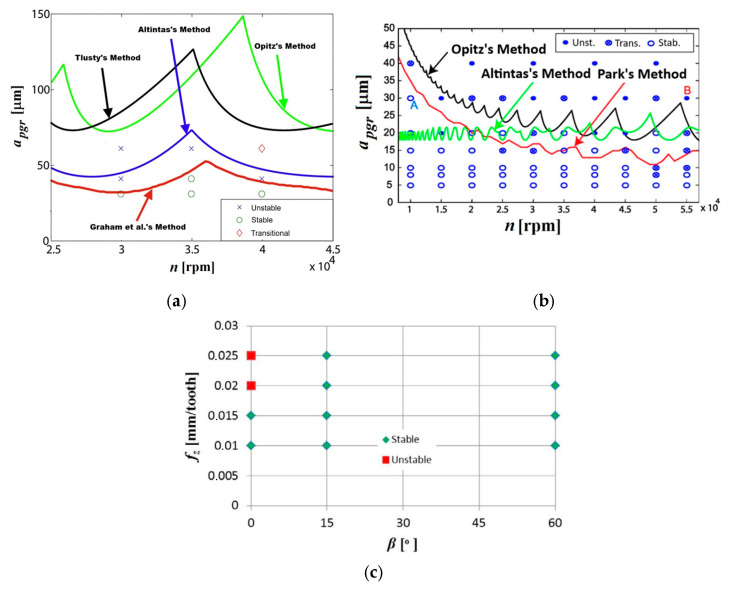
Stability lobe charts developed for micromilling processes: (**a**) Graham et al., (**b**) Park and Rahnama, (**c**) Wojciechowski and Mrozek. Developed on the basis of [126,128,148].

During micro-milling, the frequency of self-excited vibrations usually corresponds to the tool’s natural frequencies [97,136]. Therefore, the identification of the dominant component of the vibration spectrum, corresponding to the tool’s natural frequency, enables the recognition of the unstable machining area. The studies conducted by Uhlmann and Mahr [136] show that the presence of self-excited vibrations can also be confirmed by microscopic SEM analysis of the surface after micro-milling. In the case of unstable end milling, the machining marks of self-excited vibrations (chatter marks) form the so-called Moiré patterns. This may indicate the superposition of two different frequencies during the mapping of the tool in the workpiece material (e.g., the self-excited vibration frequency and the tooth passing frequency). The most popular method for assessing the dynamical stability of precision and micro machining is the analytical formulation of stability lobe diagrams (SLD) based on a dynamic deflection model [140]. Studies have shown that conventional cutting models [141,142,143] are characterized by a significant error in the case of micromilling. It should be emphasized that very high spindle rotational speeds are usually used during precision machining, which results in large gyroscopic moments and centrifugal forces [144,145]. As a result, this may affect the differences in the obtained spindle natural frequencies depending on the rotational speed. Certain phenomena characteristic of precision machining may affect the fluctuation of forces and thus the variability of the proportionality coefficients in the analytical model. Graham et al. [126,132] proposed an analytical stability model for precision milling, which takes into account the variability of the spindle natural frequency and the proportionality coefficients in the force equations. The authors assumed that the variation in the proportionality coefficients results from the elastic recovery of the material and the two-phase microstructure of the workpiece. It has been shown that the developed stability model for precision milling shows better agreement with the experimental results compared to the effects obtained for traditional stability models (Figure 9a).

According to Park and Rahnama [128], the damping force plays an important role in the dynamic stability of the micro-milling process. During precision machining with small uncut chip thicknesses, the elastic recovery of the workpiece material is observed, leading to an increase in the coefficient of friction on the tool flank face, and thus to an increase in the damping force [146]. Taking into account the aforementioned force (described by Equation (18)) in the analytical stability model leads to an increase in the critical (stable) depth of cut in the range of lower spindle speeds (Figure 9b).

Another significant factor that can affect the stability of machining process in a diversified way is the ploughing phenomenon. Typically, the ploughing appears when the workpiece material is being subjected to an intense elastic and plastic deformations occurring for a range of *h_z_* < *h*_min_. Consequently, it can lead to a growth of instant forces acting along the curved cutting edge of a tool. The aforementioned growth of forces can be attributed to both the growth of a ploughing volume between the tool flank face and workpiece (which appears in a range of low uncut chip thickness values), as well as the uncut chip thickness accumulation phenomenon, which can induce the local growth of uncut chip thickness [118]. The growth and high variability of force values, together with a phenomenon of transition from ploughing to shearing, can lead to the excitation of one or more natural frequencies of the machining system and thus to the stability loss [120]. On the other hand, the intensification of a ploughing phenomenon (manifesting by the growth of a ploughing volume) can lead to a growth of a contact length between the tool flank face and workpiece, which in turn can induce a growth of friction coefficient and thus the damping force [147]. In turn, an increase in damping force can affect dynamic stability growth of machining. According to Wojciechowski and Mrozek [148], in slot micro ball end milling (process without tool axis or workpiece inclination), the stability loss is being observed during machining with a higher selected feed per tooth values (Figure 9c). On the other hand, during micro ball end milling with the inclined tool axis (angle *β* > 0), the process remains dynamically stable for all the tested feed per tooth values. This stability loss for slot micromilling conditions (*β* = 0) could be due to the intense ploughing phenomenon (resulting from a very low cutting speeds in a vicinity of the tool tip), which in turn could intensify the chip thickness accumulation.

The precise milling conducted on industrial robots (robotic milling) is also the process in which the process dynamics has a critical role. It is related mainly to a specific dynamical properties of industrial robots related to their susceptibility to generating excessive vibrations and variable forces during various manufacturing processes [149,150,151,152]. Therefore, in robotic precise milling, three interrelated phenomena must be addressed: vibration phenomena and process stability, as well as cutting-force prediction.

In terms of cutting-force prediction, the significant compliance in an articulated robot’s kinematic chain alters the instantaneous chip cross-section and thus the force equilibrium at the tool–workpiece interface. Cen and Melkote [153] demonstrated that coupling a multi-body dynamic model with mechanistic force relations reduced prediction errors by over 50% compared to rigid-body assumptions, especially at high feeds and depths of cut. The authors also analyzed the significance of the effect of robot dynamics on the resulting forces as a function of robot configuration and cutting condition. Building on this, Cvitanic et al. [154] presents a comparative study of robot pose optimization using static and dynamic stiffness models for different cutting scenarios. The authors introduced pose-dependent stiffness maps to calibrate specific cutting coefficients as functions of joint configuration, yielding force predictions within 10% of experimental measurements across typical machining conditions. Sample-based planning approaches further automate this process: Diaz Posada et al. [155] generated motion trajectories that maximize structural stiffness via sampling-based planners, thereby minimizing force fluctuations without manual tuning. The optimal motion is computed based on the semantic and mathematical interpretation of the manufacturing process modeled on its components. Liao et al. [156] optimized both robot posture and workpiece setup by enforcing a stiffness threshold, significantly improving force consistency and reducing tool deflection in five-axis milling operations. This work constructs a minimum set covering problem, which is solved by a clustering algorithm and a greedy algorithm. More recently, Mun et al. [157] introduced an indirect cutting-force estimation method for robotic milling by fusing signals from a spindle-mounted accelerometer and a flange-mounted capacitive force sensor through a machine-learning-based system identifier. Their approach achieved prediction errors below 15% against dynamometer measurements across a range of feeds and depths of cut, enabling real-time adaptive adjustment of cutting parameters. 

The inherent flexibility of industrial robots also gives rise to vibration phenomena that critically affect surface quality and tool life. Bisu et al. [158] performed a comprehensive dynamic behavior analysis of a six-axis machining robot, identifying multiple low-frequency structural modes (below 80 Hz) that dominate the unloaded response and can be excited during cutting. The study [159] reveals that low-frequency structural resonances in a si*x*-axis robot’s arm—excited during light aluminum milling are the primary source of vibrations that degrade surface finish. Through experimental modal testing in two poses selected for maximum and minimum static stiffness, the authors show that it is the resonant peaks in dynamic compliance, not static stiffness alone, that determine vibration amplitude and the resulting roughness. To capture this, they propose the Oriented Dynamic Compliance metric, which describes the direction-dependent dynamic response of the tool tip under cutting forces. Ultimately, the work demonstrates that effective robot-pose optimization must blend both static and dynamic criteria to suppress vibrations and enhance the quality of the machined surface. The study [160] reports the integration of an active inertial actuator into a robotic milling spindle to boost dynamic stiffness and suppress low-frequency structural vibrations that impair machining performance and surface quality. Authors identify the actuator’s model parameters in both horizontal and vertical mountings and design tailored compensation filters model based for the vertical configuration and pole-zero placement for the horizontal setup achieving reductions in the robot’s low frequency resonant modes by approximately 100% and 214%, respectively. In earlier work, Pan and Zhang [161] establish cutting force and robot structure models to systematically dissect chatter in robotic milling, revealing that, unlike the regenerative chatter typical of CNC machines, robotic machining is dominated by mode-coupling chatter due to the robot’s low structural stiffness. They then derive stability criteria, experimentally validate them on a six-DOF robot, and offer practical guidelines for process setup and cutting-parameter selection to achieve chatter-free robotic milling.

Finally, process stability and chatter must be tackled through predictive modeling, pose optimization, and adaptive control. In work [162], the authors propose an in-process method for predicting the frequency response function (FRF) by combining experimental FRFs measured at various robot poses with Gaussian Process Regression (GPR). Based on this, they determine process stability (via stability lobe diagrams) accounting for variations in feed rate and robot orientation and validate their approach through time domain simulations and milling tests. Cvitanic et al. [154] conduct a comparative study of robot-pose optimization in milling by developing both static stiffness models (ignoring mass and damping) and dynamic stiffness models (including mass and damping). Their time-domain milling experiments show that dynamic model-based pose selection significantly reduces end effector deflections and suppresses vibrations when the cutting force frequency content approaches the robot’s natural frequencies, whereas static model-based optimization is adequate when those frequencies are located away from the robot’s resonance frequencies.

In addition to the prediction of vibrations in the stable and unstable conditions, dynamic deflection models are applied to predict the surface topography and surface roughness after precision machining. Wang et al. [46] proposed a model for surface topography prediction including cutting edge motion expressions and dynamics of thin-walled parts. Mathematical equations were applied to characterize the tool tip trajectory, concerning tool and workpiece vibrations. Chen et al. [49] elaborated a three-dimensional surface topography model for micromilling, concerning process kinematics, run-out, and nonlinear dynamics. Based on validation, it has been presented that the model accurately predicts machined surface topographies both in stable and unstable micromilling conditions. Miao et al. [50] included a workpiece and tool dynamics expressions into a surface topography model intended for peripheral milling processes. The authors have found that vibrations generated in the tool–workpiece system have an influential effect on the shape and amplitude of surface topography formed during milling. Yuan et al. [51] modeled 3D surface topography during precise flank milling, concerning multi-order mode-dominated forced vibrations of the milling tool. Authors observed that forced vibrations affect surface roughness heights and the geometry of milling marks.

## 5. Generalized Approaches for Force and Vibrations Prediction

**Figure 10:**
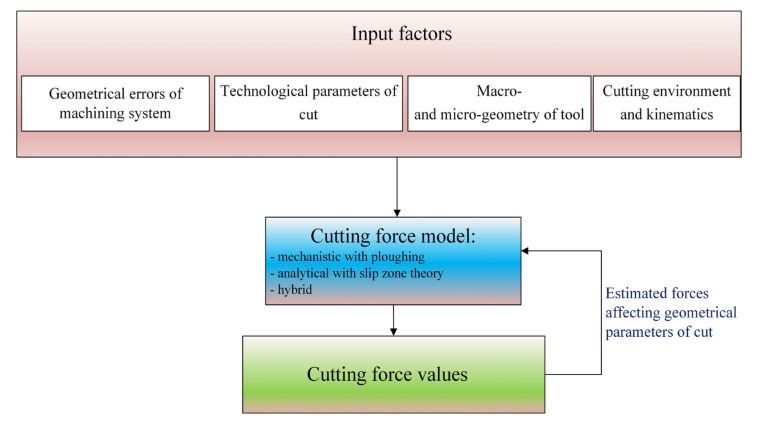
Generalized cutting force model for precise and micromilling processes.

**Figure 11:**
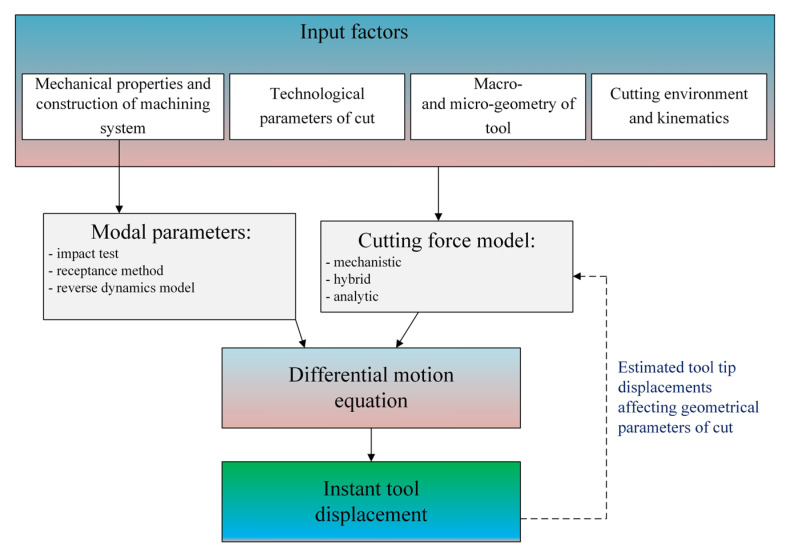
Generalized vibrations model for precise and micromilling processes.

The presented force and vibrations models for precision and micromilling differ significantly from the approaches used in conventional machining. The advantage in the accuracy of force and vibrations estimation in the case of precision machining models increases with the decrease in the uncut chip thickness to cutting edge radius *h*/*r_n_* ratio. This results from the occurrence of the so-called size effect in precision and micro machining and the significant influence of instant displacements in the machining system on the variability of the geometric parameters of cut (e.g., uncut chip thickness and uncut chip width). Based on the literature review, a generalized scheme for a modeling of forces and vibrations in precision machining can be formulated (Figure 10 and Figure 11). Among the input parameters of the precision machining process, the geometric errors of the machining system become significant, determining the value of the radial and axial run-outs (and thus the uncut chip thickness, and uncut chip width), as well as the microgeometry of the cutting edge taking into account the cutting edge radius *r_n_*.

A very important aspect is the type of force model, which should take into account phenomena occurring in the range *h* ≈ *h*_min_ (e.g., ploughing, elastic recovery of the material, strain hardening, and the uncut chip thickness accumulation). Therefore, among the forms of force models, modified mechanistic models, analytical models taking into account the slip zone, and hybrid models have been used. Finally, in the case of highly flexible milling cutters (e.g., micromills), the calculation algorithm should also take into account tool deflections caused by the impact of forces. Therefore, the forces estimated at the initial stage of modeling are used to determine the geometric parameters of cut, and at a later stage to re-determine the force values. However, it should be noted that the majority of force models concerning the force–tool tip deflection relationships include the static deflection models, which significantly simplify and shorten the derivations. 

On the other hand, in the case of vibrations models intended for a precise and micromilling processes the force–tool tip deflection relationships are being calculated on the basis of dynamics models incorporating the modal parameters of the machining system (Figure 11). The necessity to use dynamics models results from the need to accurately estimate machining system displacements in a wide range of rotational speeds, as well as the ability to determine the stability limit of the system.

**Figure 12:**
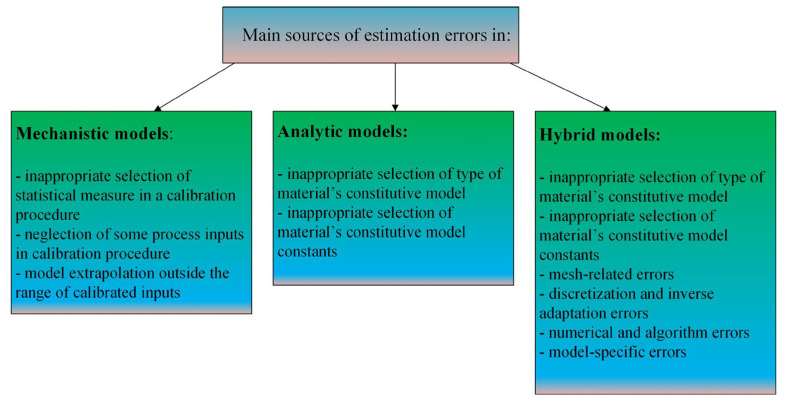
Characterization of main sources of estimation errors in modeling procedures.

The force and displacement/vibrations models presented in this study can be successfully employed to estimate the physical phenomena appearing during precise and micromilling processes of various workpiece materials and in the range of various process inputs. However, like all simulation methods applied in science, they are subject to estimation errors. Independently on the modeling procedure, the estimation errors can be affected by some random factors, such as variations in the structure, properties and surface finish of the workpiece, as well as the presence of a transient vibrations in machining system (e.g., related to a condition of a machine tool and/or tool input and output). On the other hand, some sources of errors are determined and strictly related to the structure of a specified modeling method (Figure 12).

In the case of mechanistic approaches, the main sources of estimation errors can be related to a specific cutting force calibration procedure. The specific cutting force coefficients can be estimated based on calculations, which consider the various statistical measures of the registered experimental forces (e.g., the peak and pit force values, the mean arithmetic values, and others). Consequently, the selected calibration approach can affect the conformity of the modeled signal’s peak and pit values with the experimental ones. The specific cutting force coefficients’ calibration procedure is conducted for a selected machining parameters and in a specified range of process inputs. Therefore, applying the mechanistic model in a range of variable inputs (e.g., various cutting speeds), which were not considered in a calibration procedure, can induce significant modeling errors. In addition, since the calibration procedure is being conducted for a selected range of machining inputs, the model’s extrapolation outside the calibrated inputs can contribute to a growth of estimation errors (especially for a machining inputs, which affect the machining process non-monotonically, such as cutting speed or uncut chip thickness).

Concerning the analytical approaches, the main sources of estimation errors are related to a proper selection of a workpiece material’s constitutive model and its constants. The main problem here is associated with possible differences in the properties of reference materials (for which the model constants were determined) and ones applied in real experiments. In this context, even a slight change in the material’s mechanical properties and structure can affect the values of a constitutive model constants and thus the estimation errors. Moreover, some difficulties in application of analytic models can be related to multi-phase and novel composite materials for which the values of constants are unavailable or the traditional constitutive models are characterized by insufficient accuracy.

In case of hybrid approaches, some sources of estimation errors are the same as for analytic models, since they are based on constitutive material models. However, the hybrid models are most often based on the finite element (FE) methods, the specificity of which may also be a significant source of estimation errors. In this context, the sources of estimation errors can be originated from the mesh-related errors (e.g., mesh density and quality, element type and size) [163], discretization and inverse adaptation errors [164,165], numerical and algorithm errors [166,167], as well as the model-specific errors (e.g., geometric and material properties, boundary and loading conditions) [168,169]. 

Concluding, the estimation errors in aforementioned modeling methods are affected by many different factors and the specificity of modeling procedures. However, based on the conducted literature review, it can be noted that independently on the type of applied model and selected process inputs, the estimation errors are usually within the range of between 5% and 20%. 

## 6. Conclusions and Outlook

This paper has been focused on the critical literature review of a recent studies devoted to the prediction of cutting forces and vibrations during precise and micromilling operations. To that end, almost 170 relevant research and review papers concerning the subjects of modeling and experimentation of forces and vibrations were thoroughly evaluated.

The analysis shows that force models for precision machining differ significantly from conventional approaches. These differences result from the need to take into account the rounded cutting edge radius, geometric errors of the machining system, the impact of the size effect, and also, in the case of slender tools, the effect of tool deflection on the cutting force. Many authors have proposed the analytical models with slip zone theory and hybrid force models based on the simultaneous use of analytical or experimental methods and finite element methods (FEM). Using the latter approach enables significant simplification of the complex and time-consuming calculations related to the thermomechanical phenomena appearing during the chip formation processes.

Much attention is also paid to the displacements of the machining system elements. Both static and dynamic deflection models are taken into account in the works on this issue. The use of the above-mentioned methods allows for the estimation of vibrations in the process, assessment of dynamic stability, and prediction of the surface topography. However, most of the developed tool displacement models are based only on the deflection model. On the other hand, in the field of precision machining, the resultant displacements of the tool tip can also be significantly determined by geometric errors of the machining system.

The conducted review reveals a significant role of force–displacement interactions in the aforementioned modeling approaches. The generated cutting forces can have an influential effect on the slender tool tip displacements/vibrations, which in turn affect the instant area of cut and thus again the force values. Therefore, the prediction methods usually consider the force–displacement feedback in the proposed models, which from one side improves the accuracy of estimation, but from a second side could inhibit and extend the calculations.

The conducted analysis from a recent study enabled the identification of an existing research gaps within this scope. In a majority of the studies, the minimum uncut chip thickness is selected as a constant value during the whole CWE cycle. Nevertheless, the value of this parameter can be variable along the active length of cutting edge (especially for a torus and ball end mills), which results from distinct thermomechanical conditions in various parts of a cutting edge during CWE cycle. In addition, the real values of a cutting edge radius can exhibit some influential variations along the cutting edge, which additionally could lead to a minimum uncut chip thickness variations. It is therefore justified to conduct further research on estimating the variability of the minimum uncut chip thickness for different cutting methods and tools with different cutting edge contours, especially in the context of modeling forces and vibrations. 

In the conducted research, little attention is paid to the significant influence of cutting temperature, as well as the properties and microstructure of workpiece on the force–vibration interactions. In case of the first factor, the temperature-induced tool tip displacements (especially for micromilling tools) can influentially affect the values of geometrical elements of cut (especially cutting width) and, in this way, the values of cutting forces and vibrations. Concerning the properties and microstructure of a workpiece, their variations/fluctuations (especially hardness, tensile strength, diversification of grain size, etc.) can significantly affect the values of a specific force coefficient or chip formation mechanism during machining with very low uncut chip thicknesses, and therefore the values of forces and vibrations. Thus, the novel and accurate models concerning the diversification of workpiece structure and thermal interactions in a machining system could be developed.

The state of the art shows that only in a few works concerning modeling of forces in the range of *h_z_* ≤ *h*_min_, the influence of ploughing and elastic recovery of the workpiece material on the actual values of the geometric parameters of cut was taken into account. Neglecting this phenomenon may affect the qualitative and quantitative errors of the estimated forces. The analysis of the literature also shows an ambiguous effect of the ploughing phenomenon on the stability of the precision and micromilling processes. According to some authors, increasing the uncut chip thickness, corresponding to the transition from ploughing to shearing, can lead to the excitation of one or more natural frequencies of the machining system, and thus contribute to the stability loss. Other studies have shown that the ploughing phenomenon and the elastic recovery of the workpiece material lead to an increase in the coefficient of friction on the tool flank face, and thus to an increase in the damping force and process stability. Therefore, the issue of the precise determination of ploughing forces, in the range of variable machining parameters, as well as the relationship between these forces and the dynamics of precision milling, requires further, comprehensive research. In this context, it is also advised to extend the research on the influence of uncut chip thickness accumulation phenomenon on the generation of process vibrations and constitution of a machined surface topography.

## Data Availability

No new data were created or analyzed in this study. Data sharing is not applicable to this article.

## References

[1] Guo Q., Liu Z., Yang Z., Jiang Y., Sun Y., Xu J., Zhao W., Wang W., Wang W., Ren Q. (2024). Development, challenges and future trends on the fabrication of micro-textured surfaces using milling technology. J. Manuf. Process..

[2] Li H., Wu B. (2016). Development of a hybrid cutting force model for micromilling of brass. Int. J. Mech. Sci..

[3] Xue B., Zhang J., Sun Q., Geng Y., Yan Y., Cui H. (2024). Diffraction characteristics and formation mechanism of nanogratings in tip-based down-milling. Int. J. Mech. Sci..

[4] Zhang Y., Li S., Zhu K. (2020). Generic instantaneous force modeling and comprehensive real engagement identification in micro-milling. Int. J. Mech. Sci..

[5] Wang D., Ren J., Tian W. (2020). A method for the prediction of cutting force for 5-axis ball-end milling of workpieces with curved surfaces. Int. J. Adv. Manuf. Technol..

[6] Denkena B., Krüger M., Bachrathy D., Stepan G. (2012). Model based reconstruction of milled surface topography from measured cutting forces. Int. J. Mach. Tools Manuf..

[7] Cui Z., Liu H., Wu L., Cao Z., Zong W. (2024). Cutting force and surface quality in ultra-precision milling of oxygen-free copper under different cutting strategies. J. Manuf. Process..

[8] Brito L.C., Gomes M.C., de Oliveira D., Bacci da Silva M., Viana Duarte M.A. (2023). Vibration features for indirect monitoring of end micromilling process. Precis. Eng..

[9] Li S., Li Y., Li Y., Chen D. (2024). Study of different cutting fluids effect on the coupling characteristics of milling noise-vibration and surface roughness of TA2 pure titanium. J. Manuf. Process..

[10] Sheikhi M.R., Gürgen S., Li J. (2024). Vibration suppression and surface quality enhancement in milling thin-walled structures using shear thickening fluids. J. Manuf. Process..

[11] Wang D., Penter L., Hänel A., Ihlenfeldt S., Wiercigroch M. (2022). Stability enhancement and chatter suppression in continuous radial immersion milling. Int. J. Mech. Sci..

[12] Zhang S.J., To S. (2013). The effects of spindle vibration on surface generation in ultra-precision raster milling. Int. J. Mach. Tools Manuf..

[13] Mann B.P., Edes B.T., Easley S.J., Young K.A., Ma K. (2008). Chatter vibration and surface location error prediction for helical end mills. Int. J. Mach. Tools Manuf..

[14] Deng D., Zhang Z., Wan W., Ma Q., Sun J. (2022). Investigation on burr formation characteristics in micro milling of Ω-shaped reentrant microchannels. J. Manuf. Process..

[15] Wu Y., Chen N., Bian R., He N., Li Z., Li L. (2020). Investigations on burr formation mechanisms in micro milling of high-aspect-ratio titanium alloy ti-6al-4v structures. Int. J. Mech. Sci..

[16] Yadav R., Chakladar N.D., Paul S. (2022). Micro-milling of Ti-6Al-4V with controlled burr formation. Int. J. Mech. Sci..

[17] Zhang X., Yu T., Wang W., Zhao J. (2019). Improved analytical prediction of burr formation in micro end milling. Int. J. Mech. Sci..

[18] Becze C.E., Clayton P., Chen L., El-Wardany T.I., Elbestawi M.A. (2000). High-speed five-axis milling of hardened tool steel. Int. J. Mach. Tools Manuf..

[19] Twardowski P., Legutko S., Krolczyk G.M., Hloch S. (2015). Investigation of wear and tool life of coated carbide and cubic boron nitride cutting tools in high speed milling. Adv. Mech. Eng..

[20] Lopez de Lacalle L.N., Lamikiz A., Sanchez J.A., Salgado M.A. (2007). Toolpath selection based on the minimum deflection cutting forces in the programming of complex surfaces milling. Int. J. Mach. Tools Manuf..

[21] Wojciechowski S., Maruda R.W., Krolczyk G.M., Nieslony P. (2017). Application of signal to noise ratio and grey relational analysis to minimize forces and vibrations during precise ball end milling. Precis. Eng..

[22] Budak E., Altintas Y., Armarego E.J.A. (1996). Prediction of milling force coefficients from orthogonal cutting data. Trans. ASME J. Manuf. Sci. Eng..

[23] Dornfeld D., Lee D.E. (2008). Precision Manufacturing.

[24] Fontaine M., Devillez A., Moufki A., Dudzinski D. (2006). Predictive force model for ball-end milling and experimental validation with a wavelike form machining test. Int. J. Mach. Tools Manuf..

[25] Fontaine M., Moufki A., Devillez A., Dudzinski D. (2007). Modelling of cutting forces in ball-end milling with tool-surface inclination. Part I: Predictive force model and experimental validation. J. Mater. Process. Technol..

[26] Chiou C.H., Hong M.S., Ehmann K.F. (2005). Instantaneous shear plane based cutting force model for end milling. J. Mater. Process. Technol..

[27] Kienzle O. (1952). Prediction of forces and power in machine tools for metal-cutting. VDI-Z.

[28] Kline W.A., DeVor R.E. (1983). The effect of runout on cutting geometry and forces in end milling. Int. J. Mach. Tool Des. Res..

[29] Kline W.A., DeVor R.E. (1982). The prediction of cutting forces in end milling with application to cornering cuts. Int. J. Mach. Tool Des. Res..

[30] Ko J.H., Cho D.-W. (2005). 3D ball-end milling force model using instantaneous cutting force coefficients. J. Manuf. Sci. Eng..

[31] Lee P., Altintas Y. (1996). Prediction of ball–end milling forces from orthogonal cutting data. Int. J. Mach. Tools Manuf..

[32] Rao V.S., Rao P.V.M. (2006). Effect of workpiece curvature on cutting forces and surface error in machining of curved geometries. Proc. Inst. Mech. Eng. Part B J. Eng. Manuf..

[33] Kim J.D., Kim D.S. (1995). Theoretical analysis of micro-cutting characteristics in ultra-precision machining. J. Mater. Process. Technol..

[34] Kim C.-J., Mayor J.R., Ni J. (2004). A static model of chip formation in microscale milling. J. Manuf. Sci. Eng..

[35] Zhang X., Yu T., Wang W. (2018). Prediction of cutting forces and instantaneous tool deflection in micro end milling by considering tool run-out. Int. J. Mech. Sci..

[36] Biró I., Szalay T. (2017). Extension of empirical specific cutting force model for the process of fine chip-removing milling. Int. J. Adv. Manuf. Technol..

[37] Yuan Y., Jing X., Ehmann K.F., Cao J., Li H., Zhang D. (2018). Modeling of cutting forces in micro end-milling. J. Manuf. Process..

[38] Liu T., Zhang K., Wang G., Wang C. (2021). Prediction of Nonlinear Micro-milling force with a novel minimum uncut chip thickness model. Micromachines.

[39] Wang P., Bai Q., Cheng K., Zhao L., Ding H. (2022). The modelling and analysis of micro-milling forces for fabricating thin-walled micro-parts considering machining dynamics. Machines.

[40] Chen N., Li L., Wu J., Qian J., He N., Reynaerts D. (2019). Research on the ploughing force in micro milling of soft-brittle crystals. Int. J. Mech. Sci..

[41] Zhou L., Deng B., Peng F., Yang M., Yan R. (2020). Semi-analytic modelling of cutting forces in micro ball-end milling of NAK80 steel with wear-varying cutting edge and associated nonlinear process characteristics. Int. J. Mech. Sci..

[42] Hao Y., Liu Y. (2017). Analysis of milling surface roughness prediction for thin-walled parts with curved surface. Int. J. Adv. Manuf. Technol..

[43] Honeycutt A., Schmitz T.L. (2017). A Study of Milling Surface Quality during Period-2 Bifurcations. Procedia Manuf..

[44] Mori T., Hiramatsu T., Shamoto E. (2011). Simultaneous double-sided milling of flexible plates with high accuracy and high efficiency suppression of forced chatter vibration with synchronized single-tooth cutters. Precis. Eng..

[45] Shan C., Lv X., Duan W. (2016). Effect of tool inclination angle on the elastic deformation of thin-walled parts in multi-axis ball-end milling. Procedia CIRP.

[46] Wang Z., Wang B., Yuan J. (2018). Modeling of surface topography based on cutting vibration in ball-end milling of thin-walled parts. Int. J. Adv. Manuf. Technol..

[47] Peng F., Wu J., Fang Z., Yuan S., Yan R., Bai Q. (2013). Modeling and controlling of surface micro-topography feature in micro-ball-end milling. Int. J. Adv. Manuf. Technol..

[48] Chen H.-Q., Wang Q.-H. (2018). Modeling and simulation of the surface topography in ball-end milling based on biharmonic spline interpolation. Int. J. Adv. Manuf. Technol..

[49] Chen W., Xie W., Huo D., Yang K. (2018). A novel 3D surface generation model for micro milling based on homogeneous matrix transformation and dynamic regenerative effect. Int. J. Mech. Sci..

[50] Miao H., Li C., Liu C., Wang C., Zhang X., Sun W. (2024). Machined surface prediction and reliability analysis in peripheral milling operations. Int. J. Mech. Sci..

[51] Yuan X., Fan Y., Liang Z., Wang S., Mao X., Xie X., Yang A., Liu H., Xu Y. (2024). Prediction of measured surface topography with forced vibration effects. Measurement.

[52] Wojciechowski S., Twardowski P. (2014). The influence of tool wear on the vibrations during ball end milling of hardened steel. Procedia CIRP.

[53] Iglesias A., Munoa J., Ciurana J., Dombovari Z., Stepan G. (2016). Analytical expressions for chatter analysis in milling operations with one dominant mode. J. Sound Vib..

[54] Zheng C.M., Junz Wang J.-J. (2013). Stability prediction in radial immersion for milling with symmetric structure. Int. J. Mach. Tools Manuf..

[55] Zhou K., Feng P., Xu C., Zhang J., Wu Z. (2017). High-order full discretization methods for milling stability prediction by interpolating the delay term of time delayed differential equations. Int. J. Adv. Manuf. Technol..

[56] Chen Q., Li W., Ren Y., Zhou Z. (2020). 3D chatter stability of high-speed micromilling by considering nonlinear cutting coefficients, and process damping. J. Manuf. Process..

[57] Singh K.K., Kartik V., Singh R. (2015). Modeling dynamic stability in high-speed micromilling of Ti-6Al-4V via velocity and chip load dependent cutting coefficients. Int. J. Mach. Tools Manuf..

[58] Singh K.K., Kartik V., Singh R. (2019). Stability modeling with dynamic run-out in high speed micromilling of Ti6Al4V. Int. J. Mech. Sci..

[59] Soori M., Arezoo B., Habibi M. (2014). Virtual machining considering dimensional, geometrical and tool deflection errors in three-axis CNC milling machines. J. Manuf. Syst..

[60] Dorgeloh T., Beinhauer A., Riemer O., Brinksmeier E. (2016). Microfluidic balancing concepts for ultraprecision high speed applications. Procedia CIRP.

[61] Peterka J., Kováč M., Zvončan M. (2011). Influence of tool balancing on machined surface quality in high speed machining. J. Prod. Eng..

[62] Saha S., Deb S., Bandyopadhyay P.P. (2021). Progressive wear based tool failure analysis during dry and MQL assisted sustainable micro-milling. Int. J. Mech. Sci..

[63] Saha S., Deb S., Bandyopadhyay P.P. (2023). Tool wear induced burr formation and concomitant reduction in MQL wetting capability in micro-milling. Int. J. Mech. Sci..

[64] Zhang C., Guo S., Zhang H., Zhou L. (2013). Modeling and predicting for surface topography considering tool wear in milling process. Int. J. Adv. Manuf. Technol..

[65] Zhang C., Zhang H., Li Y., Zhou L. (2015). Modeling and on-line simulation of surface topography considering tool wear in multi-axis milling process. Int. J. Adv. Manuf. Technol..

[66] Zhang X., Yu T., Zhao J. (2020). Surface generation modeling of micro milling process with stochastic tool wear. Precis. Eng..

[67] Jiang X., Ding J., Wang C., Hong L., Yao W., Yu W. (2024). Influence of tool wear on geometric surface modeling for TC4 titanium alloy milling. J. Manuf. Process..

[68] Wojciechowski S., Twardowski P. (2012). Tool life and process dynamics in high speed ball end milling of hardened steel. Procedia CIRP.

[69] De Aguiar M.M., Diniz A.E., Pederiva R. (2013). Correlating surface roughness, tool wear and tool vibration in the milling process of hardened steel using long slender tools. Int. J. Mach. Tools Manuf..

[70] Klocke F., Lung D., Puls H. (2013). FEM-Modelling of the thermal workpiece deformation in dry turning. Procedia CIRP.

[71] Creighton E., Honegger A., Tulsian A., Mukhopadhyay D. (2010). Analysis of thermal errors in a high-speed micro-milling spindle. Int. J. Mach. Tools Manuf..

[72] Chen W., Teng X., Huo D., Wang Q. (2017). An improved cutting force model for micro milling considering machining dynamics. 1006. Int. J. Adv. Manuf. Technol..

[73] Gozu E., Karpat Y. (2017). Uncertainty analysis of force coefficients during micromilling of titanium alloy. Int. J. Adv. Manuf. Technol..

[74] Park S.S., Malekian M. (2009). Mechanistic modeling and accurate measurement of micro end milling forces. CIRP Ann.—Manuf. Technol..

[75] Zhang X., Ehmann K.F., Yu T., Wang W. (2016). Cutting forces in micro end milling processes. Int. J. Mach. Tools Manuf..

[76] Budak E. (2006). Analytical models for high performance milling, Part I: Cutting forces, structural deformations and tolerance integrity. Int. J. Mach. Tools Manuf..

[77] Denkena B., Vehmeyer J., Niederwestberg D., Maaß P. (2014). Identification of the specific cutting force for geometrically defined cutting edges and varying cutting conditions. Int. J. Mach. Tools Manuf..

[78] Gradisek J., Kalveram M., Weinert K. (2004). Mechanistic identification of specific force coefficients for a general end mill. Int. J. Mach. Tools Manuf..

[79] Lamikiz A., López de Lacalle L.N., Sanchez J.A., Salgado M.A. (2004). Cutting force estimation in sculptured surface milling. Int. J. Mach. Tools Manuf..

[80] Ozturk B., Lazoglu I., Erdim H. (2006). Machining of free-form surfaces. Part II: Calibration and forces. Int. J. Mach. Tools Manuf..

[81] Wojciechowski S. (2015). The estimation of cutting forces and specific force coefficients during finishing ball end milling of inclined surfaces. Int. J. Mach. Tools Manuf..

[82] Grossi N., Sallese L., Scippa A., Campatelli G. (2015). Speed-varying cutting force coefficient identification in milling. Precis. Eng..

[83] Rubeo M.A., Schmitz T.L. (2016). Milling Force Modeling: A Comparison of Two Approaches. Procedia Manuf..

[84] Tsai M.Y., Chang S.Y., Hung J.P., Wang C.C. (2016). Investigation of milling cutting forces and cutting coefficient for aluminum 6060-T6. Comput. Electr. Eng..

[85] Gonzalo O., Beristain J., Jauregi H., Sanz C. (2010). A method for the identification of the specific force coefficients for mechanistic milling simulation. Int. J. Mach. Tools Manuf..

[86] Wan M., Lu M.-S., Zhang W.-H., Yang Y. (2012). A new ternary-mechanism model for the prediction of cutting forces in flat end milling. Int. J. Mach. Tools Manuf..

[87] Zhang X., Zhang J., Pang B., Zhao W.-H. (2016). An accurate prediction method of cutting forces in 5-axis flank milling of sculptured surface. Int. J. Mach. Tools Manuf..

[88] Zhou L., Peng F.Y., Yan R., Yao P.F., Yang C.C., Li B. (2015). Analytical modeling and experimental validation of micro end-milling cutting forces considering edge radius and material strengthening effects. Int. J. Mach. Tools Manuf..

[89] Srinivasa Y.V., Shunmugam M.S. (2013). Mechanistic model for prediction of cutting forces in micro end-milling and experimental comparison. Int. J. Mach. Tools Manuf..

[90] Bo L., Yanlong C., Wenhua C., Jun P. (2017). Geometry simulation and evaluation of the surface topography in five-axis ball-end milling. Int. J. Adv. Manuf. Technol..

[91] Abdelmoneim M.E.S., Scrutton R.F. (1974). Tool edge roundness and stable build up formation in finish machining. Trans. ASME J. Eng. Ind..

[92] Waldorf D.J., DeVor R.E., Kapoor S.G. (1998). A slip line field for ploughing during orthogonal cutting. Trans. ASME J. Manuf. Sci. Eng..

[93] Jun B.G., Liu X., DeVor R.E., Kapoor S.G. (2006). Investigation of the Dynamics of Microend Milling. Part I: Model Development. J. Manuf. Sci. Eng..

[94] Fang N. (2003). Slip-Line Modeling of Machining With a Rounded-Edge Tool. Part I: New Model and Theory. J. Mech. Phys. Solids.

[95] Afazov S.M., Ratchev S.M., Segal J., Popov A.A. (2012). Chatter modelling in micro-milling by considering process nonlinearities. Int. J. Mach. Tools Manuf..

[96] Afazov S.M., Ratchev S.M., Segal J. (2010). Modelling and simulation of micro-milling cutting forces. J. Mater. Process. Technol..

[97] Afazov S.M., Zdebski D., Ratchev S.M., Segal J., Liu S. (2013). Effects of micro-milling conditions on the cutting forces and process stability. J. Mater. Process. Technol..

[98] Jin X., Altintas Y. (2012). Prediction of micro-milling forces with finite element method. J. Mater. Process. Technol..

[99] Lai X.M., Li H.T., Li C.F., Lin Z.Q., Ni J. (2008). Modeling and analysis of micro scale milling considering size effect, micro cutter edge radius and minimum chip thickness. Int. J. Mach. Tools Manuf..

[100] Jin X., Altintas Y. (2011). Slip-line field model of micro-cutting process with round tool edge effect. J. Mater. Process. Technol..

[101] Karpat Y. (2009). Investigation of the effect of cutting tool edge radius on material separation due to ductile fracture in machining. Int. J. Mech. Sci..

[102] Liu K., Melkote S. (2007). Finite element analysis of the influence of tool edge radius on size effect in orthogonal micro-cutting process. Int. J. Mech. Sci..

[103] Liu K., Melkote S. (2006). Material strengthening mechanisms and their contribution to size effect in micro-cutting. J. Manuf. Sci. Eng..

[104] Ding H., Shen N., Shin Y.C. (2011). Experimental evaluation and modeling analysis of micromilling of hardened H13 tool steels. J. Manuf. Sci. Eng..

[105] Zhou Y., Tian Y., Jing X., Ehmann K.F. (2017). A novel instantaneous uncut chip thickness model for mechanistic cutting force model in micro-end-milling. Int. J. Adv. Manuf. Technol..

[106] Luo S., Jun M.B.G., Dong Z. (2016). Numerical Simulation of Chip Ploughing Volume and Forces in 5-axis CNC Micro-milling Using Flat-end Mills. Procedia Manuf..

[107] Bao W.Y., Tansel I.N. (2000). Modeling micro-end-milling operations. Part I: Analytical cutting force model. Int. J. Mach. Tools Manuf..

[108] Li H.Z., Liu K., Li X.P. (2001). A new method for determining the undeformed chip thickness in milling. J. Mater. Process. Technol..

[109] Perez H., Vizan A., Hernandez J.C., Guzman M. (2007). Estimation of cutting forces in micromilling through the determination of specific cutting pressures. J. Mater. Process. Technol..

[110] Urbikain G., Artetxe E., López de Lacalle L.N. (2017). Numerical simulation of milling forces with barrel-shaped tools considering runout and tool inclination angles. Appl. Math. Model..

[111] Zhu K., Zhang Y. (2017). Modeling of the instantaneous milling force per tooth with tool run-out effect in high speed ball-end milling. Int. J. Mach. Tools Manuf..

[112] Zhu Z., Yan R., Peng F., Duan X., Zhou L., Song K., Guo C. (2016). Parametric chip thickness model based cutting forces estimation considering cutter runout of five-axis general end milling. Int. J. Mach. Tools Manuf..

[113] Omar O.E.E.K., El-Wardany T., Ng E., Elbestawi M.A. (2007). An improved cutting force and surface topography prediction model in end milling. Int. J. Mach. Tools Manuf..

[114] Sastry S., Kapoor S.G., DeVor R.E. (2000). Compensation of progressive radial run-out in face-milling by spindle speed variation. Int. J. Mach. Tools Manuf..

[115] Desai K., Agarwal P.K., Rao P.V.M. (2009). Process geometry modelling with cutter runout for milling ofcurved surfaces. Int. J. Mach. Tools Manuf..

[116] Moges T.M., Desai K.A., Rao P.V.M. (2016). Improved Process Geometry Model with Cutter Runout and Elastic Recovery in Micro-End Milling. Procedia Manuf..

[117] Rodríguez P., Labarga J.E. (2013). A new model for the prediction of cutting forces in micro-end-milling operations. J. Mater. Process. Technol..

[118] Wojciechowski S., Matuszak M., Powałka B., Madajewski M., Maruda R.W., Królczyk G.M. (2019). Prediction of cutting forces during micro end milling considering chip thickness accumulation. Int. J. Mach. Tools Manuf..

[119] Sutherland J.W., DeVor R.E. (1986). Improved method for cutting force and surface error prediction in flexible end milling systems. J. Eng. Ind..

[120] Jun B.G., DeVor R.E., Kapoor S.G. (2006). Investigation of the Dynamics of Micro end Milling. Part II: Model Validation and Interpretation. J. Manuf. Sci. Eng..

[121] Budak E., Altintas Y. (1995). Modeling and avoidance of static deformations in peripheral milling of plates. Int. J. Mach. Tool Des. Res..

[122] Kops L., Vo D.T. (1990). Determination of the equivalent diameter of an endmill based on its compliance. Ann. CIRP.

[123] Kivanc E., Budak E. (2004). Structural modeling of end mills for form error and stability analysis. Int. J. Mach. Tools Manuf..

[124] Kim G.M., Kim B.H., Chu C.N. (2003). Estimation of cutter deflection and form error in ball-end milling processes. Int. J. Mach. Tools Manuf..

[125] López de Lacalle L.N., Lamikiz A., Sanchez J.A., Salgado M.A. (2004). Effects of tool deflection in the high-speed milling of inclined surfaces. Int. J. Adv. Manuf. Technol..

[126] Graham E., Mehrpouya M., Park S.S. (2013). Robust prediction of chatter stability in milling based on the analytical chatter stability. J. Manuf. Process..

[127] Insperger T., Gradisek J., Kalveram M., Stepan G., Winert K., Govekar E. (2006). Machine Tool Chatter and Surface Location Error in Milling Processes. J. Manuf. Sci. Eng..

[128] Park S.S., Rahnama R. (2010). Robust chatter stability in micro-milling operations. CIRP Ann.—Manuf. Technol..

[129] Wang J.J., Uhlmann E., Oberschmidt D., Sung C.F., Perfilov I. (2016). Critical depth of cut and asymptotic spindle speed for chatter in micro milling with process damping. CIRP Ann.—Manuf. Technol..

[130] Rahnama R., Sajjadi M., Park S.S. (2009). Suppression of Chatter in Micro Milling with Process Damping. J. Mater. Process. Technol..

[131] Wojciechowski S., Tabaszewski M., Krolczyk G.M., Maruda R.W. (2017). The study on dynamic properties of monolithic ball end mills with various slenderness. E3S Web Conf..

[132] Graham E., Mehrpouya M., Nagamune R., Park S.S. (2014). Robust prediction of chatter stability in micro milling comparing edge theorem and LMI. CIRP J. Manuf. Sci. Technol..

[133] Park S.S., Altintas Y., Movahhedy M. (2003). Receptance coupling for endmills. Int. J. Mach. Tools Manuf..

[134] Eynian M. (2015). Prediction of vibration frequencies in milling using modified Nyquist method. CIRP J. Manuf. Sci. Technol..

[135] Yılmaz E.E., Budak E., Özgüven H.N. (2016). Modeling and Measurement of Micro End Mill Dynamics Using Inverse Stability Approach. Procedia CIRP.

[136] Uhlmann E., Mahr F. (2012). A Time Domain Simulation Approach for Micro Milling Processes. Procedia CIRP.

[137] Yuan M., Wang X., Jiao L., Yi J., Liu S. (2017). Prediction of dimension error based on the deflection of cutting tool in micro ball-end milling. Int. J. Adv. Manuf. Technol..

[138] Mamedov A., Layegh K.S.E., Lazoglu I. (2013). Machining forces and tool deflections in micro milling. Procedia CIRP.

[139] Matuszak M., Kochmański P., Powałka B., Ševĉik L., Lepšík P., Petrů M., Mašín I., Martonka R. (2014). Workpiece Grain Size Influence on the Vibration in Micro-milling. Modern Methods of Construction Design.

[140] Mittal R.K., Kulkarni S.S., Singh R.K. (2017). Effect of lubrication on machining response and dynamic instability in high-speed micromilling of Ti-6Al-4V. J. Manuf. Process..

[141] Altintas Y., Weck M. (2004). Chatter Stability of Metal Cutting and Grinding. Ann. CIRP.

[142] Opitz H. (1970). Investigation and calculation of the chatter behavior of lathes and milling machines. Ann. CIRP.

[143] Tlusty J. (2000). Manufacturing Processes and Equipment.

[144] Jorgensen B.R., Shin Y.C. (1998). Dynamics of spindle bearing systems at high speeds including cutting load effects. J. Manuf. Sci. Eng..

[145] Movahhedy M.R., Mosaddegh P. (2006). Prediction of chatter in high speed milling including gyroscopic effects. Int. J. Mach. Tools Manuf..

[146] Altintas Y., Eynian M., Onozuka H. (2008). Identification of Dynamic Cutting Force Coefficients and Chatter Stability with Process Damping. Ann. CIRP.

[147] Haung C.Y., Junz Wang J.J. (2007). Mechanistic Modeling of Process damping in peripheral milling. ASME J. Manuf. Sci. Eng..

[148] Wojciechowski S., Mrozek K. (2017). Mechanical and technological aspects of micro ball end milling with various tool inclinations. Int. J. Mech. Sci..

[149] Cernohlavek V., Klimenda F., Houska P., Suszyński M. (2023). Vibration Measurements on a Six-Axis Collaborative Robotic Arm—Part I. Sensors.

[150] Meller A., Suszynski M., Legutko S., Trączyński M., Cernohlavek V. (2023). Studies on a Robotised Process for Forging Steel Synchronizer Rings in the Context of Forging Tool Life. Manuf. Technol..

[151] Klimenda F., Cizek R., Suszyński M. (2022). Measurement of a Vibration on a Robotic Vehicle. Sensors.

[152] Suszyński M., Wiśniewski M., Wojciechowicz K., Trączyński M., Butlewski M., Cernohlavek V., Talar R. (2025). Study of Positioning Accuracy Parameters in Selected Configurations of a Modular Industrial Robot—Part 1. Sensors.

[153] Cen L., Melkote S.N. (2017). Effect of Robot Dynamics on the Machining Forces in Robotic Milling. Procedia Manuf..

[154] Cvitanic T., Nguyen V., Melkote S.N. (2020). Pose Optimization in Robotic Machining Using Static and Dynamic Stiffness Models. Robot. Comput.-Integr. Manuf..

[155] Diaz Posada J.R., Schneider U., Sridhar A., Verl A. (2017). Automatic Motion Generation for Robotic Milling Optimizing Stiffness with Sample-Based Planning. Machines.

[156] Liao Z.-Y., Wang Q.-H., Xie H.-L., Li J.-R., Zhou X.-F., Pan T.-H. (2022). Optimization of Robot Posture and Workpiece Setup in Robotic Milling with Stiffness Threshold. IEEE/ASME Trans. Mechatron..

[157] Mun C.H., Rezvani S., Lee J., Park S., Park H.W., Lee J. (2022). Indirect measurement of cutting forces during robotic milling using multiple sensors and a machine learning-based system identifier. J. Manuf. Process..

[158] Bisu C.-F., Cherif M., Gérard A., K’Nevez J.-Y. (2012). Dynamic Behavior Analysis for a Six-Axis Industrial Machining Robot. arXiv.

[159] Leonesio M., Villagrossi E., Beschi M., Marini A., Bianchi G., Pedrocchi N., Molinari Tosatti L., Grechishnikov V., Ilyukhin Y., Isaev A. (2018). Vibration analysis of robotic milling tasks. Procedia CIRP.

[160] Raparelli Sahu G.N., Otto A., Ihlenfeldt S. (2024). Improving Robotic Milling Performance through Active Damping of Low-Frequency Structural Modes. J. Manuf. Mater. Process..

[161] Pan Z., Zhang H. Analysis and Suppression of Chatter in Robotic Machining Process. Proceedings of the 2007 International Conference on Control, Automation and Systems (ICCAS).

[162] (2023). Kenan Deng, Dong Gao, Chang Zhao, Yong Lu, Prediction of in-process frequency response function and chatter stability considering pose and feedrate in robotic milling. Robot. Comput.-Integr. Manuf..

[163] Rainsberger R.B., Fong J.T., Marcal P.V. Effect of mesh quality in finite element analysis of crack-tip stresses in a circumferential surface crack of a pipe elbow weldment. Proceedings of the ICF 2017—14th International Conference on Fracture.

[164] Zhuang X., Heaney C., Augarde C. (2012). On error control in the element-free Galerkin method. Eng. Anal. Bound. Elem..

[165] Dheeravongkit A., Shimada K. (2007). Inverse adaptation of a Hex-dominant mesh for large deformation finite element analysis. CAD Comput. Aided Des..

[166] Kannan R., Hendry S., Debney P. (2018). What Is Your Structural Model Not Telling You? Finding Hidden Modelling Errors and Inaccuracies in Your Analysis Results. Structures Congress 2018: Buildings and Disaster Management, Proceedings of the Structures Congress 2018, Fort Worth, TX, USA, 19–21 April 2018.

[167] Navarro-Jiménez J.M., Nadal E., Ródenas J.J. (2025). Recovery-based accuracy assessment of numerical analysis results in linear elasticity. A code for moving least squares recovery based on physics. Adv. Appl. Mech..

[168] Sankararaman S., Ling Y., Shantz C., Mahadevan S. Fatigue crack growth analysis under uncertainty. Proceedings of the 51st AIAA/ASME/ASCE/AHS/ASC Structures, Structural Dynamics and Materials Conference.

[169] Wang J.D., Howard I.M. (2006). Error analysis on finite element modeling of involute spur gears. J. Mech. Des..

